# Participatory modeling meets African swine fever – Systems Thinking in action

**DOI:** 10.1186/s12917-025-04747-3

**Published:** 2025-05-02

**Authors:** Lisa Rogoll, Katja Schulz, Jana Schulz

**Affiliations:** Institute of Epidemiology, Friedrich-Loeffler-Institut, Federal Research Institute for Animal Health, Südufer 10, 17493 Greifswald – Insel Riems, Germany

**Keywords:** African swine fever, Participatory modeling, Systems thinking, Wild boar, Stakeholder involvement, Causal loop diagram, Germany

## Abstract

**Background:**

Despite concerted efforts to control the disease, African swine fever (ASF) continues to spread in numerous regions across Europe. The circulation of the virus in wild boar populations poses an ongoing risk of transmission to domestic pig holdings and results in significant socio-economic losses. Controlling ASF in wild boar has proven to be a complex endeavor that affects many different sectors of society, with different focal points of interest and potential for conflict. To obtain a comprehensive understanding of stakeholder challenges and identify leverage points for effective interventions, a multidisciplinary Systems Thinking approach was applied. Participatory modeling workshops were conducted in an ASF-affected area of Germany with stakeholders from public institutions, forestry, conservation, agriculture, and the food industry in order to provide a comprehensive understanding of the stakeholders’ perception of the complex system of ASF control. Causal Loop Diagrams were developed jointly with stakeholders to capture and visualize the interconnections within the system from stakeholders’ perspective.

**Results:**

During the workshops, participants emphasized the need for transparent and rapid communication among all affected stakeholders and the importance of of raising public awareness about ASF for an effective response to outbreaks. Participants identified the complexity of the ASF control system, represented by a preliminary Causal Loop Diagram (CLD) with 260 loops, including 131 balancing and 129 reinforcing loops. These feedback processes illustrate the dynamic and interconnected nature of ASF control efforts, highlighting both challenges and opportunities for intervention. In addition, the participants stressed the need for early preparation of relevant resources for potential ASF outbreaks. Effectiveness and unintended consequences of control measures represented a major concern for the participants. Furthermore, participants emphasized the need to standardize and harmonize processes across different regions of Germany to improve the effectiveness of ASF control efforts.

**Conclusion:**

The findings underscore the importance of integrating stakeholder insights into the design of ASF control measures to ensure their effectiveness and sustainability. The complexity of the system, as represented by the numerous feedback loops, highlights the need for adaptive and collaborative approaches in managing ASF outbreaks. Moving forward, discussions with scientists and decision-makers will be essential to refine and implement these insights into actionable strategies for effective ASF management. Engaging stakeholders in the modeling process fosters ownership, collaboration, and compliance, which are crucial for successful implementation of ASF control measures. Furthermore, transdisciplinary research is providing valuable insights for regions or countries yet unaffected by ASF, helping them prepare effectively for potential outbreaks.

**Supplementary Information:**

The online version contains supplementary material available at 10.1186/s12917-025-04747-3.

## Background

African swine fever (ASF) is a viral disease originating from the African continent that has emerged widely throughout Europe and beyond in the last decade [1–3]. The virus can cause severe hemorrhagic disease with high lethality in various suid species including domestic pigs and Eurasian wild boar [4].

The first case of ASF in wild boar in Germany was reported in September 2020 in the north-eastern part of Germany, close to the Polish border [5]. Since then, the disease has spread in parts of the wild boar populations of the federal states Brandenburg, Saxony and Mecklenburg-Western Pomerania, posing a potential risk of disease transmission to domestic pig holdings. Despite great efforts to control the disease, ASF continues to spread in the wild boar populations of certain regions of Germany [6]. Recently, in the summer of 2024, the virus spread to a previously unaffected area further away from the initial epidemic front in the east, marking a significant expansion of the outbreak. Consequently, the federal states of Hesse and Rhineland-Palatinate, located in western Germany, confirmed the first occurrence of ASF in wild boar [7], again highlighting the risk of indirect transmission of ASF via contaminated fomites or waste over long distances [8]. By the end of 2024, eighteen ASF outbreaks in domestic pigs were notified in Germany.

Following the Animal Health Law of the European Union, various control measures are implemented in Germany, including the reduction of the wild boar population through intensified hunting, establishment of restriction zones, fencing of affected areas as well as conducting carcass searches and sampling of wild boar. Implementing these measures requires financial and human resources and the compliance of multiple stakeholders. Furthermore, trade restrictions are in place causing devastating economic losses in the pig industry [9] and negative impacts on animal welfare of both domestic pigs and wild boar can be observed.

Consequently, diverse perspectives and interests need to be considered to maintain effective epidemic preparedness over long time periods. Thus, holistic problem-solving and decision-making approaches, such as Systems Thinking (ST), are needed. Systems Thinking is a collection of approaches that views complex phenomena as interconnected systems rather than isolated components [10]. It emphasizes understanding the relationships, feedback loops, and dynamic behavior within a system to identify underlying patterns and causes of problems. One powerful tool of the ST approach is mediated modeling [11]. In this collaborative problem-solving approach, groups of stakeholders are involved to jointly create and analyze models of complex systems. Through this process, models are developed that integrate the unique perspectives and knowledge of various stakeholders, weaving different viewpoints together into a comprehensive whole [11]. This process fosters social learning, where stakeholders acquire knowledge, refine their understanding, and potentially adapt their perspectives through interaction and shared experiences within the group. Causal loop diagrams (CLDs) visualize mental models of stakeholders, highlighting causality and feedback loops [12]. Quantitative stock and flow diagrams (SFDs) can be used to illustrate potential consequences of different decisions and strategies in a simulation model [10], ensuring preparedness for a range of potential outcomes. Including multiple perspectives and transdisciplinary knowledge can increase the legitimacy and acceptance of the models and resulting decisions and ensures preparedness for a range of potential outcomes [13, 14]. Agyepong et al. emphasized the critical importance of considering the impact of policy decisions and understanding stakeholder perceptions for effective decision making in dynamic settings [15]. To do so, ST and mediated modeling can provide a practical solution in human and animal health to improve the integration of health issues into policy planning [16, 17]. Thus, the ST approach has witnessed a surge in interest recently and has been applied in the context of health concerns and zoonotic outbreaks in several countries [18–22]. Ouma et al. also provide an example of applying ST to assess the impact of ASF interventions in peri-urban pig value chains in Uganda [23].

By implementing an ST approach in Germany in a workshop format in 2023, we aimed to provide insights into the complex processes of ASF control efforts in an affected area of Germany. By considering factors such as different farm management systems, on-farm biosecurity, wildlife interactions and socio-political factors, this approach allows to obtain a comprehensive understanding of the mental model of involved stakeholders. Furthermore, we aimed to assess the interdependencies and potential unintended consequences of different ASF control measures in Germany from the stakeholders’ perspective.

## Methods

### Study area and recruitment of participants

Six relevant sectors with different groups of stakeholders engaged in the event of an ASF outbreak were identified purposively for our study (Table 1). Eligile participants were from federal states that were affected by ASF at the time of the study (Mecklenburg-Western Pomerania, Brandenburg and Saxony) and were selected based on their interest in ASF control and willingness to participate voluntarily.

**Table 1:** Sectors of ASF control identified by the authors and respective stakeholders of each sector that were considered relevant and invited for the study

Sector	Stakeholders
Public Institutions	• Veterinary Authorities • Hunting Authorities • Forestry Authorities • State Diagnostic Laboratories
Forestry and Hunting	• Forestry rangers • Professional and private hunters and their associations
Nature and Animal Protection	• Animal welfare associations • Nature conservation associations
Agriculture	• Farmers and their associations
Food Industry	• Pig farmers • Private veterinarians • Animal health services • Abattoirs
Private Individuals and Volunteers	• Hikers • Dog walkers • Mushroom pickers • Volunteer Fire Departments • Technical Relief Organizations

Recruitment took place from December 2022 to January 2023, with invitations sent via email. A maximum of 15 participants per workshop was set to ensure the feasibility of the workshop format. In total, 93 stakeholders/institutions were invited. In addition, a random selection of 58 out of 329 hunters, who had voluntarily provided their contact information in a previous questionnaire study were contacted [24].Sampling methods varied by sector. In the sectors “Public Institutions”, “Nature and Animal Protection” and “Private Individuals and Volunteers”, counties were randomly selected and relevant local stakeholders were contacted. Representatives from the “Agriculture” and “Food Industry” sectors were selected via convenience sampling, while state diagnostic laboratories, animal health services and national animal welfare and nature conservation organizations were contacted directly [24].

All invited stakeholders were informed of the background, scope, and objectives of the study in the invitation letter.

### Workshop series

A series of three daylong workshops were held at intervals of one month each with the same group of participants in Greifswald, Insel Riems from February to April 2023. All workshops were conducted by a team of three facilitators in German language and were audio-recorded for documentational purposes. Personal characteristics of the workshop team according to the checklist of Tong et al. [25] are reported in Supplementary Table 1, Additional File 1. The workshop team combined facilitator roles as described by Richardson and Andersen between exercises [26]: For each exercise, one person acted as facilitator, the second person acted as modeler/reflector and third person acted as both process coach and recorder [26].

The workshops were designed to provide an introduction into systems thinking and modeling, by doing exercises to build up the model variables and components step by step. For this purpose, different methodologies were combined in the workshops. On the one hand, there were short lectures on the background of ASF, systems thinking and modeling. On the other hand, there were individual exercises, interactive group exercises, and group discussions, all of which were prepared with guidelines beforehand. Most of the exercises were adapted from guidelines in “Scriptapedia” [27], a collection of exercises for group model building. One guideline was prepared by the authors (see Additional File 2).

**Fig. 1:**
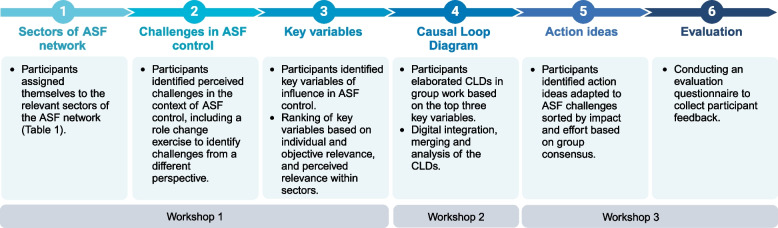
Flowchart illustrating the process and content of the participatory modeling workshops conducted in 2023 in Greifswald, Germany. There were 15 participants in workshop 1, 12 participants in workshop 2 and 9 participants in workshop 3. Created in BioRender. Rogoll, L. (2025) https://BioRender.com/v84k689

Figure 1 provides an overview of the process and contents of the participatory modeling workshops, which are described in detail in the following sections.

#### Sectors of ASF network

Participants were asked to assign themselves to the sector(s) of the ASF network (Table 1) to which they felt they belonged. Selection of more than one sector was possible.

#### Challenges in ASF control

An exercise based on the guideline "Encountered challenges" (designed by authors, Additional File 2) was used to collect challenges encountered by stakeholders in the context of ASF control. In the first part of the exercise, participants were asked to change roles with another participant and name challenges they had encountered or that they could imagine encountering regarding ASF control in their new role. Participants were then asked to name challenges they encountered or could imagine to encounter in their own role. The challenges were collected and thematically coded after the workshops using the software ATLAS.ti [28]. Main themes were derived from the coding items.

#### Key variables

The Scriptapedia exercise “Graphs over Time” [27, 29, 30] was used to identify and collect key variables that appear to be relevant in the context of ASF control and could be used to measure the success or failure of ASF control. Participants were asked to visualize the behavior of every variable over time as a graph in groups of two. Results were presented one graph at a time. The variables were afterwards compared pairwise in a pairwise ranking as described by Gay et al. [31] according to personal importance (i.e. importance for the individual) and objective importance (i.e. importance for the whole system of ASF control) by each participant individually.

Participants were then randomly assigned to a sector and were asked to identify which of the identified key variables are relevant in ASF control in their allocated sector and to add any further elements of ASF control they considered relevant in that sector. The “Think-Group-Share” principle was applied by adapting the Scriptapedia exercise “Nominal Group Technique” [27, 32–34]. First, every participant wrote down relevant key variables and additional elements individually. Afterwards, participants who collected key variables and elements for the same sector discussed their choices in small groups. Finally, the results were presented by a representative of each group and discussed with all participants, whereby eventually additional elements were added.

The individual pairwise rankings were evaluated as described by Gay et al. [31] by the workshop team. Three rankings were excluded from the analysis. One participant did not provide a complete ranking but stated that several key variables were equally important, while two others provided clearly erroneous responses (e.g., ranking only key variables 5 and 6 while stating that '10' was the most important). Since our method requires complete and meaningful rankings for valid comparisons, these cases were considered erroneous and excluded. The results of each individual personal and objective ranking were then combined by summing the ranks of each variable and assigning a new overall rank to each variable according to the sum. This produced an overall ranking of key variables by personal importance and another overall ranking of key variables by objective importance.

In addition, the variables were ranked according to the frequency with which they were selected as relevant in the sectors of the ASF network. The highest number of mentions corresponded to the highest rank. In this way, a third overall ranking was created.

Results of all three rankings (based on individual and objective relevance, and perceived relevance within sectors) were combined by summing the ranks of the respective top three key variables in order to identify the three most important variables.

#### Causal loop diagram

A short lecture was given to introduce participants to the principles of constructing and interpreting Causal Loop Diagrams (CLDs) [10]. Participants then formed small groups and collaboratively developed CLDs following the Scriptapedia exercise "Initiating and Elaborating a Causal Loop Diagram" [27, 29, 33, 35, 36], with each group focusing on one of the three most significant variables. The group assignments were randomized. Each group was guided by a facilitator and aimed to incorporate the additional key variables and elements that had been previously gathered, as well as to identify and include new elements. The CLDs were then presented to the group and explained by the facilitator.

The three CLDs were digitized and merged by using recurrent elements of the CLDs as connecting points by the workshop team using the software “iModeler” [37]. All variables, connections and polarities elaborated by the participants were thereby preserved. Duplicate connections were removed. Abbreviated connections were marked with a dashed line. The joined CLD was presented and discussed with the participants.

After the workshops, the CLD was analyzed using insight matrices generated by the “iModeler” software. The insight matrix analyzes the impact of all or selected factors on a selected target factor in the CLD. The matrix results from the sum of all effects across all impact paths that a factor has on the selected target factor. The sum of the effects can have a positive or a negative value, however, it is not an absolute value. It reflects the impact of a factor in relation to other factors. The calculated impact of factors may change over the short, medium, and long term due to the influence of balancing or reinforcing loops. Balancing loops work to stabilize the system, counteracting changes and returning it to equilibrium, while reinforcing loops amplify changes, leading to exponential growth or decline. These loops can cause the impact of factors to shift from a positive to a negative value or vice versa [38].

#### Action ideas

By adapting the Scriptapedia exercise “Action Ideas” [27, 39] to the challenges of ASF control, a collection of action ideas was generated and prioritized based on the model. In this process, the action ideas were classified according to their impact and the effort required to implement them. This approach enables a systematic evaluation of the action ideas and aids in determining which ideas should be given priority in addressing the challenges of ASF control. In a two by two grid, the action ideas were placed in the suiting quadrant according to the group consensus. The quadrants were: (I) High impact/Low effort, (II) High impact/High effort, (III) Low impact/Low effort, (IV) Low impact/High effort. If an action idea could not be clearly assigned to one quadrant, it was placed between quadrants.

#### Closing and evaluation

Adapting the Scriptapedia exercise “Next Steps and Closing” [27], the workshops were closed by giving a brief review of the series and a short outlook on the next steps. A short questionnaire to evaluate the workshop series was conducted at the end of the workshop series. The questionnaire was adapted from the list in Appendix 3, Van den Belt [11] (Supplementary Table 2, Additional File 1). Statements were ordered according to the average score, from most agreement to most disagreement: 5 = strongly agree; 4 = agree somewhat; 3 = neutral; 2 = disagree somewhat; 1 = strongly disagree.

## Results

### Participants

Initially, the group consisted of 15 people that represented hunting authorities (4 people), state diagnostic laboratories (2 people), animal and nature conservation organizations (2 people), state forestry (2 people), agriculture (2 people), veterinary authorities (1 person), zoological gardens (1 person) and private hunters (1 person). Out of this, 9 participants were female and 6 participants were male. The estimated average age of participants was between 35 to 45 years. Due to personal reasons 3 people dropped out of the study after the first workshop and 3 more people were not able to attend the third workshop.

When assigning themselves to one or more of the suggested sectors ASF control, 14 participants assigned themselves to the sector of private individuals and volunteers. Nine participants assigned themselves to the sectors of public institutions and forestry and hunting, seven to nature and animal protection, six to agriculture and five to food industry.

### Challenges in ASF control

**Fig. 2:**
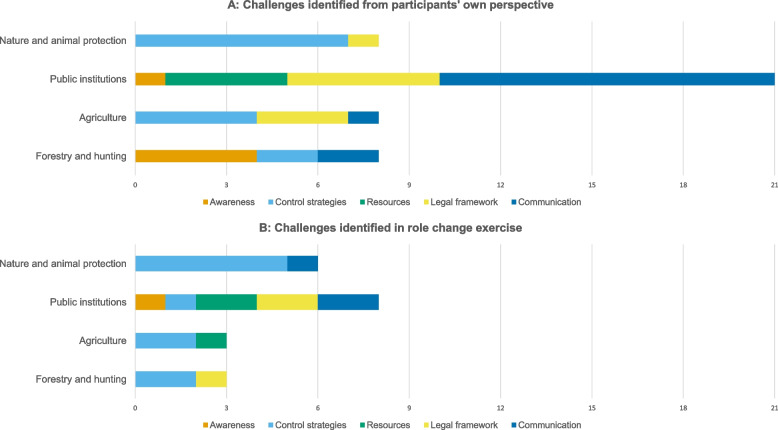
Perceived challenges within different sectors identified from participants own perspective (**A**) and from a role change exercise, where participants imagined undertaking a role different from their own (**B**) in a participatory modeling workshop conducted in 2023 in Greifswald, Germany

Sixty five challenges faced by participants related to ASF control were indentified by participants. Out of these, 45 challenges were indentified from participants own perspective and 20 challenges were identified during the role change exercise, where participants imagined to take a role different from their own during ASF control. The collected challenges mainly related to the themes of communication (*n* = 6), resources (*n* = 7), control strategies (*n* = 23), legal frameworks (*n* = 12) and awareness (*n *= 6) (Table 2). Several ideas were repeatedly mentioned by participants, both during the role change and the collection of challenges from their own perspective (Supplementary Table 3 and Supplementary Table 4, Additional File 1). Most challenges were collected in the sector of public institutions, for both own perspective and role change (Fig. 2). From their own perspectives, participants collected most challenges regarding the topic of communication (Fig. 2A). During the role change, participants collected most challenges regarding the topic of control strategies (Fig. 2B).

**Table 2:** Overview of key challenges in ASF control and thematic coding of stakeholder perspectives. The table presents the main topics and motives of challenges in regard to ASF control as reported by participants across various sectors

Topic	Codes	Explanation
Awareness	Media reports	Media reports must be technically correct and understandable
	Motivation	Long-term motivation of involved stakeholders to fight ASF and implement measures is needed
	Unknowing population	There is ignorance or lack of understanding among the general population
Control strategies	Alternative control strategies	Alternative control measures should be researched and applied
	Compulsory stabling	Compulsory stabling impedes animal welfare and has a negative impact on farmed pigs
	Effect of fences	The construction of fences has negative effects on other wildlife species
	Trade restrictions	Due to ASF restrictions, trade with pigs is only possible to a limited extent or not at all
	Timber marketing restrictions	Due to ASF restrictions, timber marketing or trade is limited or not possible at all
	Reduction of wild boar population	The reduction of the wild boar populations is a challenge
	Reasonableness	The meaningfulness and reasonableness of control measures is questionable
	Animal husbandry	Animal husbandry must be species-appropriate and in compliance with animal welfare standards
	Killing of healthy animals	The killing of healthy domestic and wild boar is ethically rejected
	Loss gene pool	Due to ASF restrictions, there will be a loss in the gene pool of certain rare pig species
Legal framework	Data management	Data collection and storage (compliant with data protection) is a challenge
	Heterogeneity of regulations	Different regulations apply in different counties/federal states
	Samples according to specifications	Samples must be submitted *(to the lab)* in accordance with official requirements, otherwise evaluation is only possible with delays or not at all
	Implementation of legal requirements	Legal regulations, guidelines, etc. are interpreted differently ("strictly") in some cases
	Different areas of law	Different areas of law are affected (animal protection, animal disease law, hunting law, property law, etc.)
Communication	Improved communication	More transparent communication among involved actors is needed, appropriate communication channels must be established
	Coordination	Clear and transparent coordination of different actors is challenging but necessary
	Preparation	Preparation for possible outbreaks is necessary, e.g. provision of personnel and establishment of a crisis team
	Cooperation	Cooperation between different actors is necessary or must be encouraged
Resources	Skills	Personnel needs sufficient expertise
	Ammunition	Availability of sufficient ammunition for hunting
	Lack of resources	Lack of personnel, technical equipment, financial resources, or the like
	Time management	Time management in the context of ASF control is a challenge

### Key variables

In total, participants identified twelve key variables (Table 3), which were categorized into three of the five thematic codes. No key variables were assigned to the topics legal framework and communication.

**Table 3:** Overview of key variables identified by the stakeholders and thematic coding. The table presents key variables that appear to be relevant in the context of ASF control and could be used to measure the success or failure of ASF control

Topic	Key variable
Awareness	Accurate media coverage
Control strategies	Number of samples (nationwide)
	Proportion of positively hunted wild boar to the total number of hunted wild boar
	Number of wild boar (wild boar population size)
	Number of domestic pig holdings
	Number of positive test results
	Size of restriction zones I and II in km^2^
	Number of pig holdings in zoos
Resources	Extent of investment in vaccination research
	Number of ASF outbreak personnel
	Number of trained search tems (Human + dog)
	Number of night vision and night targeting technology in use

The participants ranked the key variables in a pairwise comparison based on individual and objective relevance, as well as perceived relevance within sectors (Supplementary Table 5, Additional File 1). In eight of the twelve evaluated rankings, the ranking according to individual relevance equaled the ranking according to objective relevance.

The top three key variables according to individual relevance were the extent of investment in vaccine research, the number of samples nationwide and the number of positive test results. The top three key variables according to objective relevance were both the number of ASF outbreak personnel and the number of positive test results (equal highest ranking), followed by the number of samples nationwide. According to relevance in the sectors of ASF control, accurate media coverage was ranked first place since it was considered relevant in every sector. The extent of investment in vaccine research and the number of ASF outbreak personnel were both ranked second place for sector relevance, since they were considered relevant in five out of six sectors.

### Causal loop diagram

According to the results of the ranking, the participants developed preliminary CLDs in three groups, each starting with one of the key variables “Number of positive test results” (Supplementary Fig. 1, Additional File 3), “Number of ASF outbreak personnel” (Supplementary Fig. 2, Additional File 3) and “Extent of investment in vaccination research” (Supplementary Fig. 3, Additional File 3). No polarity has been assigned to some of the connections because the group consensus could not be reached..

**Fig. 3:**
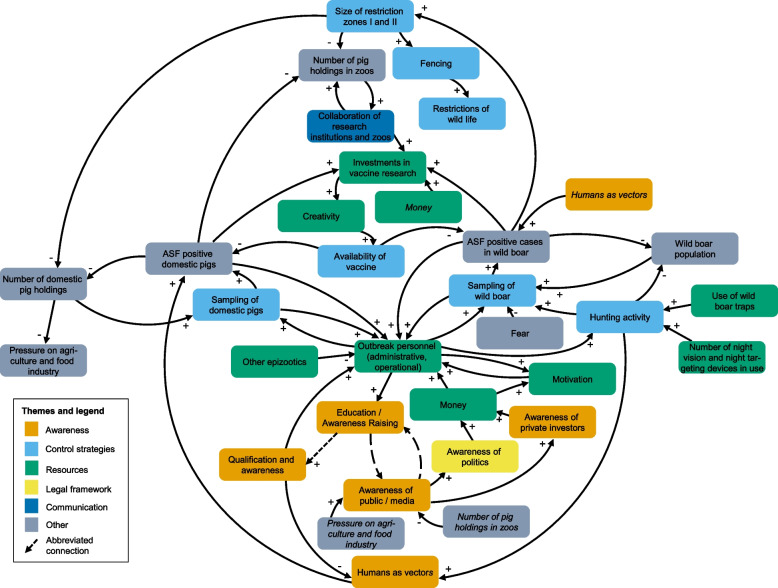
Merged Causal Loop Diagram, created jointly with participants in a participatory modeling workshop conducted in 2023 in Greifswald, Germany

Figure 3 illustrates the merged CLD from the three groups. Elements from the CLD could mostly be allocated to the main themes of control strategies, awareness, resources, legal framework and communication that were derived from previous exercises.

The CLD contains 260 loops (Additional File 4), comprising 131 balancing loops and 129 reinforcing loops. Among them, four loops contained only two elements, while three loops included 17 elements. ASF-positive domestic pigs appeared in 205 loops, whereas ASF-positive cases in wild boar were included in 224 loops. Notably, in all loops with at least 12 elements, both ASF-positive cases in wild boar and ASF-positive domestic pigs were present.

**Fig. 4:**
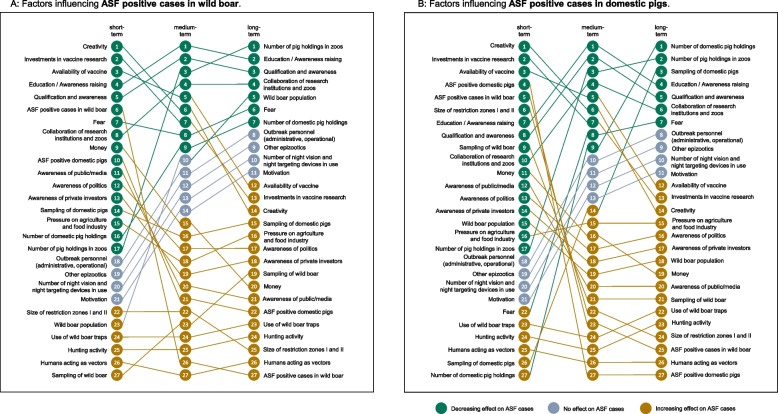
Ranking of the short-, medium- and long-term impact of factors influencing the number of ASF positive cases in wild boar (**A**) and ASF positive cases in domestic pigs (**B**) from the CLD created jointly with stakeholders in a participatory modeling workshop conducted in 2023 in Greifswald, Germany as generated by the insight matrix by the “iModeler” software [38]. The ranking of the insight matrix only allows comparison of the impact of factors in relation to each other

Insight matrices generated by the iModeler software were used to assess the short-, medium- and long-term impact of influencing factors on ASF positive cases in wild boar and ASF outbreaks in domestic pigs (Fig. 4). For both target variables 17 factors with a decreasing short-term impact, four factors with no short-term impact and six factors with an increasing short-term impact were identified. The factor impact can change over time due to loops and delays in the CLD. Thus, for both target variables seven factors with a decreasing long-term impact, four factors with no long-term impact and 16 factors with an increasing long-term impact were identified.

### Action ideas

**Fig. 5:**
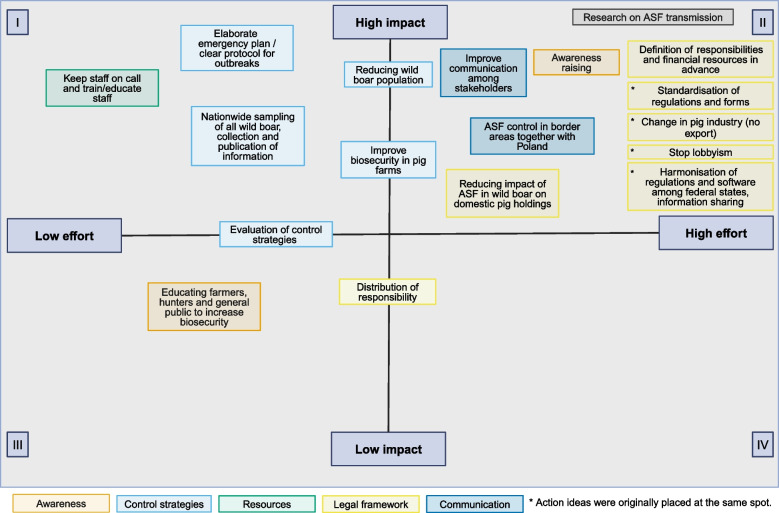
Action ideas identified and classified regarding perceived effort and impact using group consensus of stakeholders in a partipatory modeling workshop conducted in 2023 in Greifswald, Germany. Created in BioRender. Rogoll, L. (2025) https://BioRender.com/v84k689

Eighteen action ideas were identified by participants (Fig. 5). Ideas in quadrant I (high impact/low effort) were predominantly related to the topic of adjustment of control measures (light blue) and the availability of well-trained staff (green). Ideas in quadrant II (high impact/high effort) were predominantly related to improving communication and collaboration (dark blue) and amending the legal framework of ASF control (yellow) in order to harmonize control strategies across different German regions and to redefine control strategies for domestic pig farmers. One idea in that quadrant referred to increased awareness (orange). Another action idea regarding awareness raising among hunters, farmers and the general public was assigned to quadrant III (low effort/low impact).

### Evaluation

**Fig. 6:**
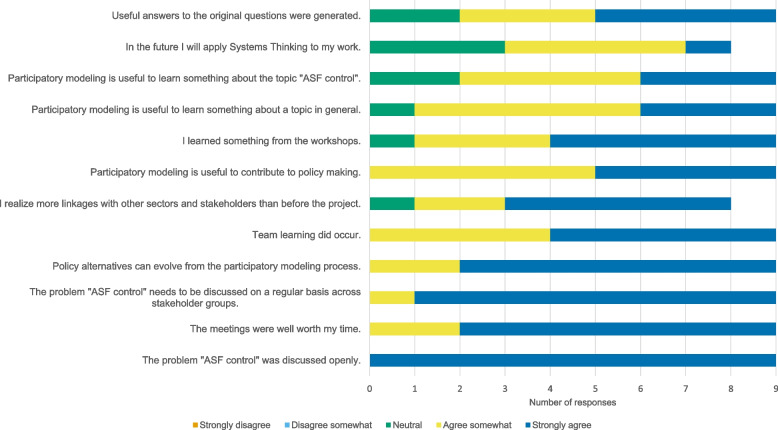
Participants’ evaluation of the participatory modeling workshop series. Only selected statements are shown, the complete results are shown in Supplementary Table 2, Additional File 1

Nine participants took part in the evaluation questionnaire on site at the end of Workshop 3 (Supplementary Table 2, Additional File 1). The other six participants were asked to complete the questionnaire online, however, no answers were received. The results of the evaluation for selected statements are shown in Fig. 6.

All participants strongly agreed that the problem of ASF control was discussed openly during the meetings. In addition, the majority of participants strongly agreed that the problems of ASF control need to be discussed on a regular basis across different stakeholder groups. Overall, the majority felt that the meetings were well worth their time. Participants mostly considered participatory modeling useful to evolve policy alternatives, to contribute to policy making and to learn something about a topic in general as well as specifically about the topic “ASF control”. Thus, participants mostly agreed that they had learned something from the workshops, that team learning had occurred and that they were more aware of linkages with other sectors and stakeholders than before the project. Mostly, participants agreed that useful answers to original questions were generated throughout the workshops. However, only about half of the participants agreed to apply ST to their work in the future.

## Discussion

Systems thinking has been increasingly applied to understand and manage complex challenges across a wide range of fields [10]. It has proven to be a powerful approach for addressing complex, interconnected challenges across various fields, including healthcare and animal disease management [16, 18, 23, 40]. By using an holistic approach that aims to understand interrelationships, feedback loops, and emergent properties within a system, ST can identify leverage points for effective interventions that involve multiple stakeholders and sectors [17]. This makes ST an ideal methodology for addressing the challenges associated with ASF control.

By bringing together stakeholders from various sectors, the workshops provided a unique platform for participants to share their perspectives, challenge assumptions, and develop a shared understanding of the problem. The list of stakeholders considered relevant for our study is comparable to the list generated by experts in the field in a World Café approach by Jori et al. [41] and aimed to include a variety of affected stakeholder groups. Although relevant institutions and companies from the food industry sector were contacted during the recruitment process, no representative could be recruited. Even though several participants declared to have experience or overlap in their professional careers with the food industry sector, perspectives from the sector may still be underrepresented in this study.

During the first workshop, the participants were invited to review challenges of ASF control from another perspective in a role change exercise aiming at bridging the understanding between the participating stakeholder groups. The number of identified challenges clearly indicates that participants found it easier to articulate arguments from their own perspectives rather than considering those of others. This highlights the critical need for transdisciplinary communication in the context of ASF control, as it is essential for raising awareness and fostering understanding of diverse viewpoints. Participants consistently emphasized the importance of transparent and rapid communication among all affected sectors and stakeholders in subsequent workshops, revealing a significant gap that needs to be addressed to improve collaboration and compliance.

As the variables presented in the current CLD were derived from the described workshop process, some of the variables, such as "money," "motivation," "fear," "fencing," and "qualification and awareness" were not explicitly defined at the time of the workshop. These terms reflect the broad, contextual input of the participants, which was valuable for understanding the system from their perspectives. However, we acknowledge that these variables may seem ambiguous, and their lack of clear definitions limits the ability to precisely measure them or track their fluctuations.

The current CLD is intended as a preliminary representation of the stakeholder group's views, and we deliberately chose not to impose our own interpretations on these variables in order to maintain the integrity of the participants' input. As we move forward with this work, we aim to refine and clarify these variables by incorporating both scientific perspectives and feedback from decision-makers and it is planed to address these clarifications in future revisions.

This points to a limitation of our study. The relatively small number of participants who took part, despite a larger number being initially contacted might introduce a selection bias that may affect the results and limit their generalizability. Although the participants who did engage provided valuable insights, the views captured may not fully represent the broader population of stakeholders. Future studies could benefit from a larger and more diverse participant pool to mitigate this potential bias and ensure that the findings are more widely applicable. As such, this CLD does not claim to be complete or definitive. Instead, it serves as a powerful and comprehensive illustration of various stakeholder perceptions within the broader context of ASF control. It allows for a shared understanding of a complex situation, which is key to fostering dialogue, reflection and social learning [42]. The evaluation of the workshop series further confirmed that team learning occurred, with participants recognizing more connections between different stakeholders and sectors.

The topic of ASF control measures was a major concern for the participants. In the current version of the CLD, which reflected the participants’ mental model, control measures such as fencing or establishment of restriction zones are lacking potential effects on ASF case numbers. Instead, the CLD emphasized the negative consequences of these measures, such as the reduction in the number of domestic pig holdings in restriction zones and the adverse impacts on wildlife due to fencing. This suggests that for affected stakeholders, the perceived negative consequences of control measures may outweigh their potential effectiveness. Indeed, a decline in the number of pig holdings, particularly concerning small-scale holders, has been observed in several countries affected by the ASF epizootic [9, 43, 44]. Furthermore, the perceived negative effects of fencing potentially outweighing its impact on ASF control have been discussed repeatedly among scientists and stakeholders [41, 45]. However, fencing has proven successful in containing the first outbreak of ASF in the Czech Republic [46] and in slowing down the eastward spread of ASF in Germany [6]. At this point, comprehensive evaluations of the effectiveness of control measures, particularly cost–benefit analyses, are required, a point also raised by participants as a potential future action. These results also highlight the necessity of science communication as stakeholders might not be aware of potentially existing research.

The lack of belief in the effectiveness of control measures by some stakeholders may indicate declining compliance and motivation among stakeholders to sustain control measures after years of intense efforts. It is crucial to address these concerns by raising awareness of the necessity for measures, effectively communicating scientific findings to targeted groups, and involving stakeholders in the planning process. Participants in the study also recognized education and awareness-raising as impactful strategies for combating ASF, as reflected in the CLD.

In addition, participants emphasized that promoting vaccine development could have a significant long-term impact on controlling ASF. Stakeholders’ optimism about vaccine development has been noted in several studies [24, 47], likely influenced by the successful use of the oral vaccine against Classical Swine Fever, which contributed to the elimination of the disease from wild boar populations in Germany [48, 49]. However, ASF vaccine development faces substantial challenges due to several knowledge gaps, including virus-host interactions, virulence genes, immune escape mechanisms, and the complexities of immune response [50]. As a result, it will likely be several years before a suitable ASF vaccine becomes available.

A repeated point of discussion during the workshops was the legal framework of ASF control provided by European legislation and national regulations.. Participants reported that the current system presents several challenges to implementing ASF control measures. These include regulatory inconsistencies across federal states, varying interpretations of legal frameworks, and the involvement of multiple legal sectors affected by ASF control. As a result, participants emphasized the need to standardize and harmonize processes across federal states to improve the efficiency and effectiveness of ASF management efforts. In this context, participants questioned the timeliness and practicality of the legal framework's goal to fully eliminate ASF in Germany. They discussed whether this objective is achievable given the current challenges in ASF control and the complexity of the regulatory landscape. [42].

The results from this study provide valuable insights into stakeholders’ perceptions of specific aspects of ASF control, including impact of control measures and variables that influence the number of positive cases. These findings will form the foundation for further participatory research, as ST emphasizes ongoing evaluation and revision through iterative cycles. The next steps in this process will include analysis and publication of results from a similar series of workshops in an area currently unaffected by ASF. In addition, the integration of the perspectives andmental models of scientists and decision makers will support the derivation of defined solution approaches for the long-term management of ASF.

## Conclusion

This study applied a ST approach to provide useful insights into the complex and ongoing challenge of ASF control in Germany. Through active stakeholder involvement and the development of CLDs, key insights emerged on ASF control dynamics, the challenges of regulatory diversity, and the critical role of interdisciplinary communication. These findings highlight the value of ST in visualizing the interconnected nature of disease control efforts and fostering shared understanding across diverse stakeholders.

The CLD effectively captures stakeholder perceptions within the broader ASF control landscape, supporting dialogue, reflection, and social learning. By engaging stakeholders in the modeling process, ST promotes ownership, collaboration, and compliance which is essential for successful implementation of ASF control measures. Moreover, this transdisciplinary approach provides actionable insights for regions or countries yet unaffected by ASF, supporting their preparedness for potential outbreaks.

Moving forward, discussions with scientists and decision-makers will be essential to define concrete steps for refining and implementing these findings. Future research will focus on developing more specific, actionable strategies and testing these insights in real-world contexts. The study highlights the need for ongoing collaboration to identify and address the critical interventions necessary for effective ASF management.

## Supplementary Information

Additional file 1: Table S1: Workshop team characteristics and relationships, based on checklist by Tong et al. [25]. Table S2: Results of evaluation questionnaire of participatory modeling workshops conducted in 2023 in Greifswald, Germany. Table S3: Perceived challenges regarding ASF control within different sectors, collected from participants in a participatory modeling workshop conducted in 2023 in Greifswald, Germany. Table S4: Perceived challenges regarding ASF control within different sectors, collected from participants in a participatory modeling workshop during the role change exercise conducted in 2023 in Greifswald, Germany. Table S5: Ranking of key variables of influence in ASF control, identified by participants in a participatory modeling workshop conducted in 2023 in Greifswald, Germany

Additional file 2: Exercise guidelines “Encountered challenges”

Additional file 3: Figure S1: Causal Loop Diagram: "Number of positive test results"as a key variable of influence in ASF control, identified by participants in a participatory modeling workshop conducted in 2023 in Greifswald, Germany. Figure S2: Causal Loop Diagram: "Number of ASF outbreak personnel" as a key variable of influence in ASF control, identified by participants in a participatory modeling workshop conducted in 2023 in Greifswald, Germany. Figure S3: Causal Loop Diagram: "Extent of investment in vaccination research" as a key variable of influence in ASF control, identified by participants in a participatory modeling workshop conducted in 2023 in Greifswald, Germany

Additional file 4: Loops identified in the CLD

## Data Availability

No datasets were generated or analysed during the current study.
