# Supplementary material for: Participatory modeling meets African swine fever – Systems Thinking in action
Source: BMC Vet Res. 2025 May 2;21:313. doi: 10.1186/s12917-025-04747-3 (PMC12046707; doi:10.1186/s12917-025-04747-3)
Supplement: Supplementary file 1 — Additional file 1: Table S1: Workshop team characteristics and relationships, based on checklist by Tong et al. [25]. Table S2: Results of evaluation questionnaire of participatory modeling workshops conducted in 2023 in Greifswald, Germany. Table S3: Perceived challenges regarding ASF control within different sectors, collected from participants in a participatory modeling workshop conducted in 2023 in Greifswald, Germany. Table S4: Perceived challenges regarding ASF control within different sectors, collected from participants in a participatory modeling workshop during the role change exercise conducted in 2023 in Greifswald, Germany. Table S5: Ranking of key variables of influence in ASF control, identified by participants in a participatory modeling workshop conducted in 2023 in Greifswald, Germany [file 12917_2025_4747_MOESM1_ESM.pdf]

Additional File 1: Supplementary Tables.

Supplementary Table 1: Workshop team characteristics and relationships (based on checklist by Tong et al. (2007)).

**Facilitator**	**KS**	**JS**	**LR**
Personal Characteristics			
**Credentials**	Veterinary Epidemiologist	Mathematician	Veterinary Epidemiologist
**Occupation**	Researcher	Researcher	Researcher
**Gender**	Female	Female	Female
**Experience/Training**	• ASF research<br>• Practitioner in Participatory Epidemiology<br>• Communication and facilitation skills	• Modelling expertise<br>• Communication and facilitation skills	• ASF research<br>• Communication and facilitation skills
Relationship with participants			
**Relationship established**<br>*Was a relationship established prior to study commencement?*			Invitations and information were sent
**Participant knowledge of the facilitator**<br>*What did the participants know about the researcher?*	• Facilitator's occupation was shared in first workshop<br>• Facilitators assigned themselves to sectors of ASF control		
**Facilitator characteristics**<br>*What characteristics were reported about the interviewer/facilitator?*	Occupation and experience (see above) was shared in first workshop		

Supplementary Table 2: Results of evaluation questionnaire of participatory modeling workshops conducted in 2023 in Greifswald, Germany. In total, 9 evaluations were collected. The table shows statements, mean and number of participants that made no specification. Statements are ordered according to the mean score, from most agreement to most disagreement: 5 = strongly agree; 4 = agree somewhat; 3 = neutral; 2 = disagree somewhat; 1 = strongly disagree. The evaluation questionnaire was adapted from the statements of Appendix 3, Van den Belt, pp. 268–269 (Van den Belt 2004).

Statement	Rating	No specification
Workshop organization and group work		
The meetings were well organized.	5.0	-
The problem “ASF control” was discussed openly.	5.0	-
The human interactions (communication) were pleasant.	4.9	-
The meetings were well worth my time.	4.8	-
Our group worked as a real team.	4.7	-
The discussions were constructive during the workshops.	4.4	-
The workshops helped in structuring thinking.	4.3	-
The participants were oriented towards a common goal.	4.3	-
Use of participatory modeling and systems thinking		
The problem "ASF control" needs to be discussed on a regular basis across stakeholder groups.	4.9	-
Policy alternatives can evolve from the participatory modeling process.	4.8	-
The workshops were of enough interest to tell others about them.	4.7	-
Team learning did occur.	4.6	-
In the future I will be thinking more about ASF control.	4.5	1
I realize more linkages with other sectors and stakeholders than before the project.	4.5	1
Participatory modeling is useful to contribute to policy making.	4.4	-
I learned something from the workshops.	4.4	-
Participatory modeling is useful to learn something about a topic in general.	4.2	-
Participatory modeling is useful to learn something about the topic "ASF control"	4.1	-
In the future I will apply Systems Thinking to my work.	3.8	1
Conclusions about the model		
I support the conclusions drawn from the model.	4.7	-
The model could apply to other systems.	4.4	2
I feel that I contributed to the design of the model.	4.4	-
The model is of enough interest to show to others.	4.3	-
Useful answers to the original questions were generated.	4.2	-
The model represents well the problem the group set out to investigate.	4.1	1
The model is a helpful tool for me in communicating some problems in the context of ASF control.	4.0	1

Supplementary Table 3: Perceived challenges regarding ASF control within different sectors, collected from participants in a participatory modeling workshop conducted in 2023 in Greifswald, Germany. The table presents key topic and thematic codes together with its frequency of occurrence in different sectors.

Topic	Codes	Forestry and hunting	Agriculture	Public institution	Animal and nature protection
**Awareness**	Media reports	0	0	1	0
	Motivation	1	0	0	0
	Unknowing population	3	0	0	0
**Control strategies**	Alternative control strategies	1	0	0	1
	Compulsory stabling	0	2	0	1
	Effect of fences	0	0	0	1
	Trade restrictions	0	2	0	1
	Timber marketing restrictions	0	0	0	0
	Reduction of wild boar population	1	0	0	0
	Reasonableness	0	0	0	0
	Animal husbandry	0	0	0	1
	Killing of healthy animals	0	0	0	2
	Loss gene pool	0	0	0	0
**Legal framework**	Data management	0	0	2	0
	Heterogeneity of regulations	0	1	0	1
	Samples according to specifications	0	0	2	0
	Implementation of legal requirements	0	2	1	0
	Different areas of law	0	0	0	0
**Communication**	Improved communication	0	1	3	0
	Coordination	0	0	2	0
	Preparation	0	0	3	0
	Cooperation	2	0	3	0
**Resources**	Skills	0	0	1	0
	Ammunition	0	0	1	0
	Lack of resources	0	0	1	0
	Time management	0	0	1	0

Supplementary Table 4: Perceived challenges regarding ASF control within different sectors, collected from participants in a participatory modeling workshop during the role change exercise conducted in 2023 in Greifswald, Germany. The table presents key topic and thematic codes together with its frequency of occurrence in different sectors.

Topic	Codes	Forestry and hunting	Agriculture	Public institution	Animal and nature protection
**Awareness**	Media reports	0	0	0	0
	Motivation	0	0	1	0
	Unknowing population	0	0	0	0
**Control strategies**	Alternative control strategies	0	0	0	0
	Compulsory stabling	0	1	0	0
	Effect of fences	0	0	0	2
	Trade restrictions	0	0	0	0
	Timber marketing restrictions	1	0	0	0
	Reduction of wild boar population	0	0	0	0
	Reasonableness	0	0	0	0
	Animal husbandry	0	1	0	2
	Killing of healthy animals	1	0	1	0
	Loss gene pool	0	0	0	1
**Legal framework**	Data management	0	0	1	0
	Heterogeneity of regulations	0	0	0	0
	Samples according to specifications	0	0	0	0
	Implementation of legal requirements	1	0	0	0
	Different areas of law	0	0	1	0
**Communication**	Improved communication	0	0	1	1
	Coordination	0	0	1	0
	Preparation	0	0	0	0
	Cooperation	0	0	0	0
**Resources**	Skills	0	1	0	0
	Ammunition	0	0	0	0
	Lack of resources	0	0	1	0
	Time management	0	0	1	0

Supplementary Table 5: Ranking of key variables of influence in ASF control, identified by participants in a participatory modeling workshop conducted in 2023 in Greifswald, Germany. Ranks according to individual relevance and objective relevance were generated through pairwise ranking of the key variables. Rank according to relevance in sectors of ASF control was generated through counting the number of sectors in which the variable was considered relevant by participants. Thereby, Rank 1 is the highest rank (most important) and Rank 12 is the lowest rank (least important).

**Key variables**	**Rank according to …**		
	**Individual relevance**	**Objective relevance**	**Relevance in sectors**
Extent of investment in vaccination research	1	4	2
Number of ASF outbreak personnel	5	1	2
Number of trained search teams (Human + dog)	4	7	9
Number of night vision and night targeting technology in use	10	10	11
Number of samples (nationwide)	2	3	4
Proportion of positively hunted wild boar to the total number of hunted wild boar	8	8	12
Accurate media coverage	9	9	1
Number of wild boar (wild boar population size)	6	5	4
Number of domestic pig holdings	11	11	4
Number of positive test results	3	1	7
Size of restriction zones I and II* in km²	7	6	7
Number of pig holdings in zoos	11	12	9
* according to Commission Implementing Regulation (EU) 2023/594			
