# Supplementary material for: Participatory modeling meets African swine fever – Systems Thinking in action
Source: BMC Vet Res. 2025 May 2;21:313. doi: 10.1186/s12917-025-04747-3 (PMC12046707; doi:10.1186/s12917-025-04747-3)
Supplement: Supplementary file 2 — Additional file 2: Exercise guidelines “Encountered challenges” [file 12917_2025_4747_MOESM2_ESM.pdf]

Encountered challenges

This script is used to identify challenges in the given context encountered by the participants at the beginning of the project. It helps to set borders of the system to investigate.

Status:

Promising practice

Primary nature of group task:

Divergent

Time

Preparation time: 15 minutes

Time required during session: 30 minutes

Follow-up time: 15 minutes

Materials

1. Paper (e.g. A5) in two colours (approx. 5-8 sheets per participant)
2. Marker pen for each participant
3. Large blank (magnet) wall or pinboard
4. Tape, magnets or pins

Inputs:

Information/list including the background (stakeholder) of the participants

Outputs:

- List of participants' challenges in the context of the project to frame the model(s) to be build
- Overall themes of challenges

Roles

- Facilitator with good group facilitation skills
- Wall-builder to cluster challenges and talk about themes
- Modeller (optional) to listen to what is mentioned, and who is able to conceptualize the early seeds of system structure
- Runner (optional) to transfer challenges from participants to wall-builder

Steps

1. Facilitator introduces the exercise – gathering challenges encountered or that the participant could imagine encountering in the context of the project. Two parts of the exercise:
    - In the first part, participants will change role with another participant and write down challenges from the perspective of this new role.
    - In the second part of the exercise, participants write down challenges from own perspective.
2. Wall-builder or runner distributes paper (colour 1) and marker pens to the participants.
3. Wall-builder is equipped with tape, magnets or pins.
4. *Part 1.* Changed perspectives
    - Facilitator explains rules for changing roles.
    - Facilitator asks participants to spend about 5 minutes thinking about what challenges there might be in the context of the project from the perspective of this

new role. The facilitator asks the participants to write one challenge per sheet of paper.
  - The facilitator asks the participants to rank the challenges from most to least important, placing the most important on top.
  - In a round-robin fashion, each participant then reads one challenge.
  - The facilitator/runner takes each challenge that the participant has read and reflects back what the participant has said for clarity. The facilitator/runner will hand off each participants' challenge to the wall-builder and return to the next participant.
  - The wall-builder will concurrently be listening to the participants and facilitator's clarifying statements. The wall-builder will then take the challenges from facilitator or runner and cluster them into thematic groups by taping/pinning them to the wall. The wall builder may arrange clusters as the script progresses to accurately capture unifying themes.
  - After each participant has had a chance to share once, the facilitator opens the floor to participants to offer additional challenges or may go around the room until everyone has shared all of their challenges.
5. Wall-builder or runner distributes paper (colour 2) to the participants.
6. *Part 2.* Own perspective
   - Facilitator introduces the 2nd part of the exercise and askes the participants to present challenges from own perspective and experience.
   - Facilitator asks participants to spend another 5 minutes thinking about the challenges they had encountered or that they could imagine encountering in the context of the project from their own perspective. Reminder: New sheet of paper for every challenge.
   - After 5 minutes, the facilitator explains that the challenges are now being collected and sorted on the pinboard/flipchart/magnetic board. Participants should name the most important challenge.
   - The wall-builder/runner takes the paper and the wall-builder arranges it on the pinboard/flipchart/magnetic board according to the thematically appropriate points already mentioned.
   - After 2-3 rounds, the facilitator stops the process. The facilitator then emphasizes that the task serves to promote understanding of different perspectives/challenges, and that the main points should now be included.
7. The wall-builder then summarizes the challenges mentioned and explains the composition of the theme blocks. In addition, the wall-builder describes whether similar or different challenges were mentioned from the changed perspectives (colour 1) than from his own perspective (colour 2).
8. The facilitator starts a feedback round to enable a short discussion: E.g. Was it difficult to change the perspective? Was it difficult to think about challenges that other stakeholders may face? Was it interesting for the participants and did the participants learned something new when listening to the challenges of others?

Evaluation Criteria:

- Participants have shared their challenges to address in the upcoming project
- Participants understand the overall themes of the challenges

Authors

Lisa Rogoll, Katja Schulz and Jana Schulz

History

The script was adapted from the script "*Hopes and Fears*" and modified to include a role change. It was first used in a group model building (GMB) session held in Germany. The purpose of the GMB was to develop a system dynamics model of African swine fever control in wild boar. The script successfully engaged stakeholders and facilitated systems thinking and group learning in practice.

Revisions

None.

References

Rogoll et al. (2025)

Notes

None.
