# Supplementary material for: Participatory modeling meets African swine fever – Systems Thinking in action
Source: BMC Vet Res. 2025 May 2;21:313. doi: 10.1186/s12917-025-04747-3 (PMC12046707; doi:10.1186/s12917-025-04747-3)
Supplement: Supplementary file 3 — Additional file 3: Figure S1: Causal Loop Diagram: "Number of positive test results"as a key variable of influence in ASF control, identified by participants in a participatory modeling workshop conducted in 2023 in Greifswald, Germany. Figure S2: Causal Loop Diagram: "Number of ASF outbreak personnel" as a key variable of influence in ASF control, identified by participants in a participatory modeling workshop conducted in 2023 in Greifswald, Germany. Figure S3: Causal Loop Diagram: "Extent of investment in vaccination research" as a key variable of influence in ASF control, identified by participants in a participatory modeling workshop conducted in 2023 in Greifswald, Germany [file 12917_2025_4747_MOESM3_ESM.pdf]

Additional File 3: Supplementary Figures.

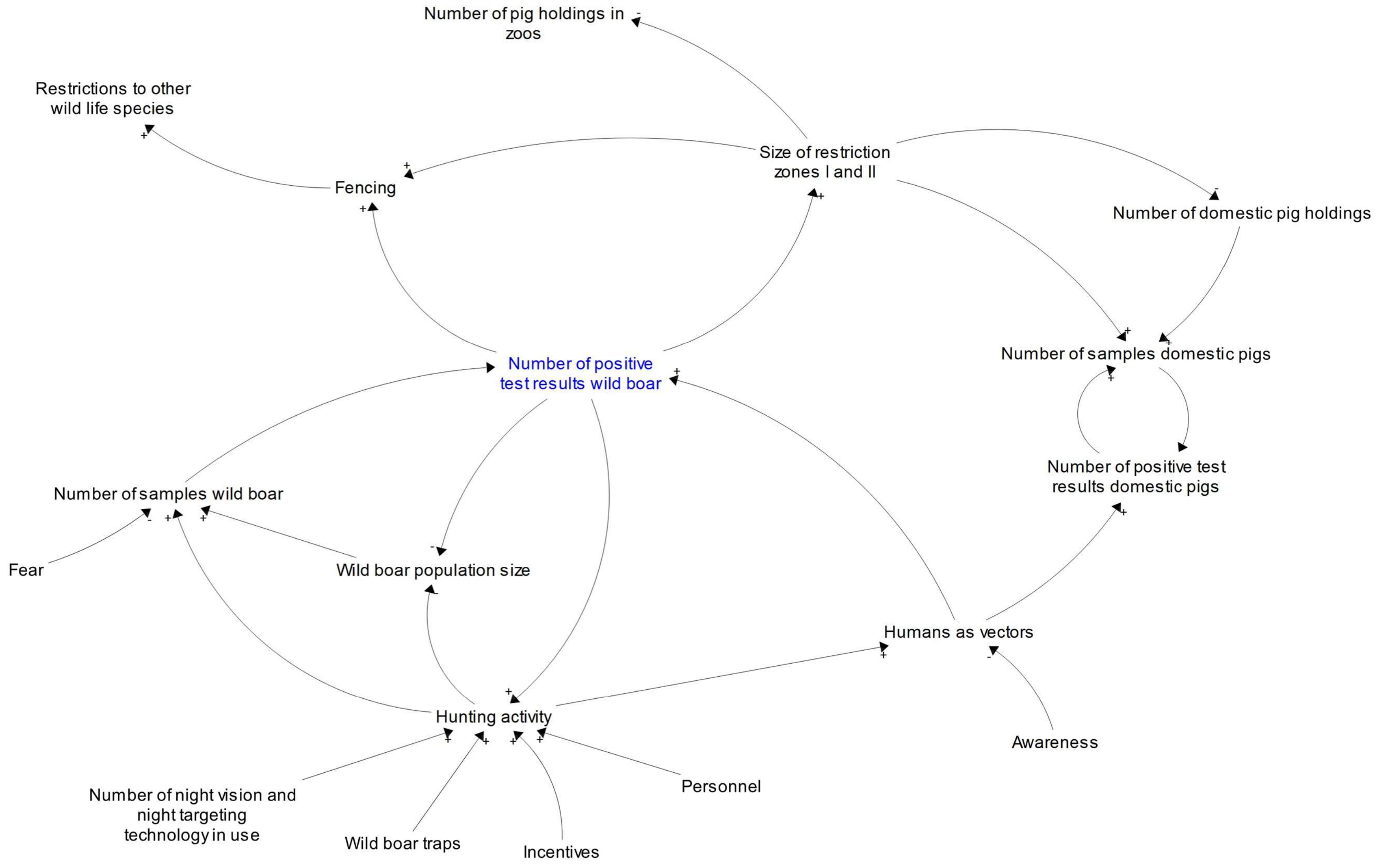

Supplementary Figure 1: Causal Loop Diagram: "Number of positive test results" as a key variable of influence in ASF control, identified by participants in a participatory modeling workshop conducted in 2023 in Greifswald, Germany. The variable was separated into "Number of positive test results in wild boar" and "Number of positive test results in domestic pigs". The group decided to start with the variable "Number of positive test results in wild boar". The figure was created using the "Stella Professional Version 3.0.1" software.

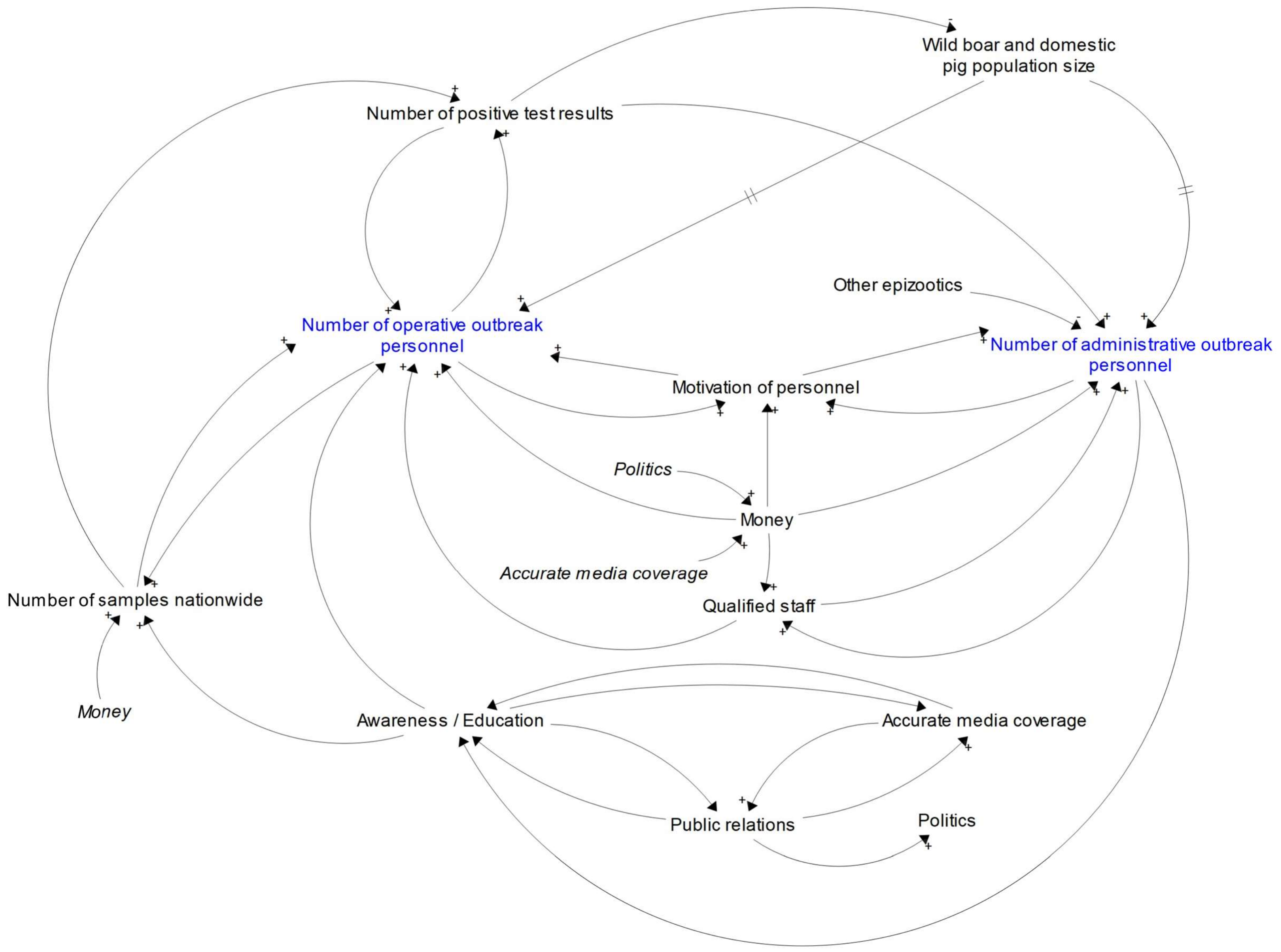

Supplementary Figure 2: Causal Loop Diagram: "Number of ASF outbreak personnel" as a key variable of influence in ASF control, identified by participants in a participatory modeling workshop conducted in 2023 in Greifswald, Germany. The group decided to divide differentiate between "Number of operative outbreak personnel" and "Number of administrative outbreak personnel". The figure was created using the "Stella Professional Version 3.0.1" software.

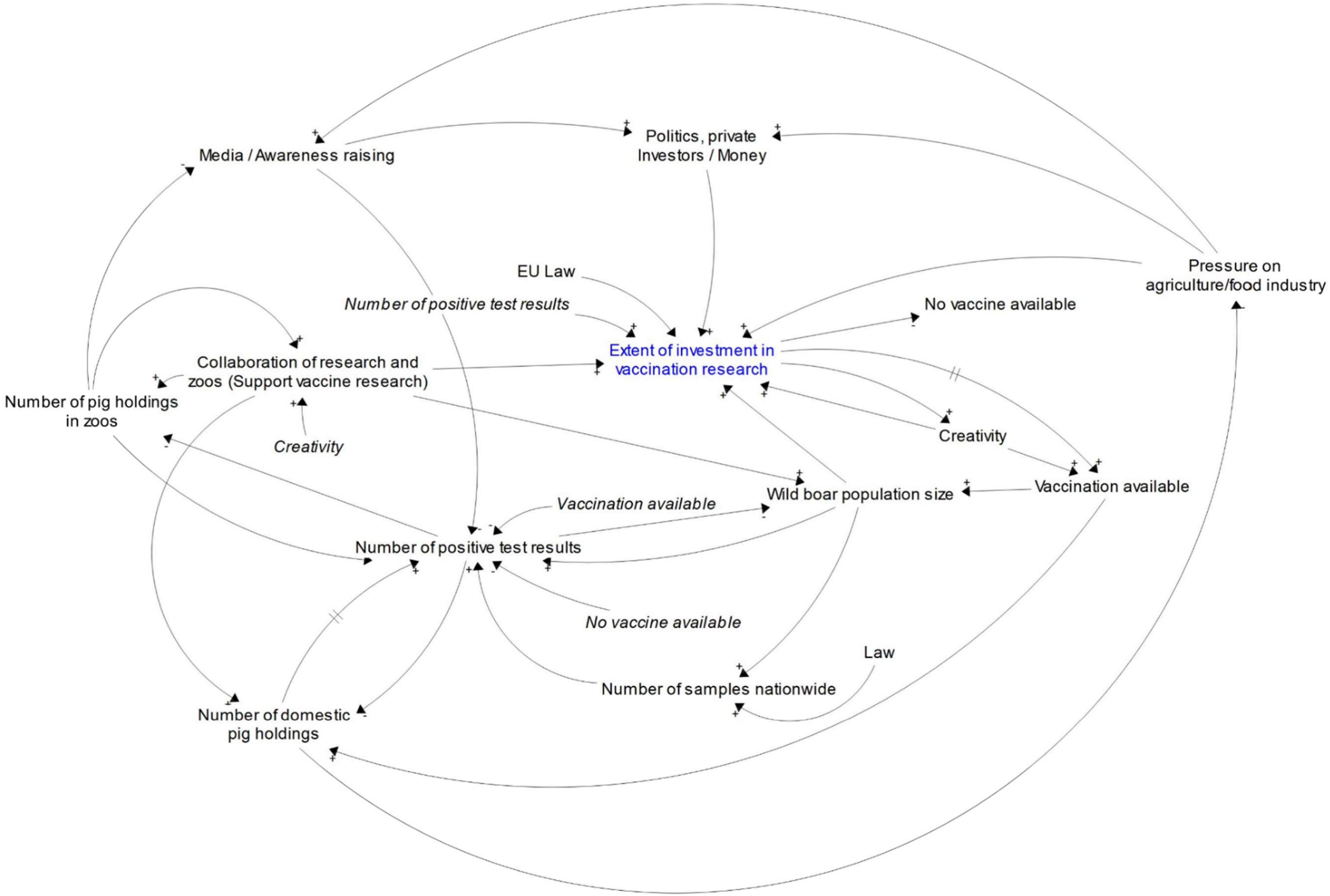

Supplementary Figure 3: Causal Loop Diagram: "Extent of investment in vaccination research" as a key variable of influence in ASF control, identified by participants in a participatory modeling workshop conducted in 2023 in Greifswald, Germany. The figure was created using the "Stella Professional Version 3.0.1" software.
