# Supplementary material for: Participatory modeling meets African swine fever – Systems Thinking in action
Source: BMC Vet Res. 2025 May 2;21:313. doi: 10.1186/s12917-025-04747-3 (PMC12046707; doi:10.1186/s12917-025-04747-3)
Supplement: Supplementary file 4 — Additional file 4: Loops identified in the CLD [file 12917_2025_4747_MOESM4_ESM.pdf]

1) **R** (2): Collaboration of research institutions and zoos -+> Number of pig holdings in zoos -+> Collaboration of research institutions and zoos

2) **R** (2): Sampling of domestic pigs -+> Outbreak personnel (administrative, operational) -+> Sampling of domestic pigs

3) **R** (2): Motivation -+> Outbreak personnel (administrative, operational) -+> Motivation

4) **R** (2): Outbreak personnel (administrative, operational) -+> Sampling of wild boar -+> Outbreak personnel (administrative, operational)

5) **B** (3): Number of domestic pig holdings -+> Sampling of domestic pigs -+> **ASF positive domestic pigs** --> Number of domestic pig holdings

6) **R** (3): **ASF positive domestic pigs** -+> Outbreak personnel (administrative, operational) -+> Sampling of domestic pigs **-+> ASF positive domestic pigs**

7) **R** (3): Education / Awareness raising -+> Qualification and awareness -+> Outbreak personnel (administrative, operational) -+> Education / Awareness raising

8) **R** (3): Outbreak personnel (administrative, operational) -+> Hunting activity -+> Sampling of wild boar -+> Outbreak personnel (administrative, operational)

9) **R** (3): Outbreak personnel (administrative, operational) -+> Sampling of wild boar -+> **ASF positive cases in wild boar** -+> Outbreak personnel (administrative, operational)

10) **B** (3): Wild boar population -+> Sampling of wild boar -+> **ASF positive cases in wild boar** --> Wild boar population

11) **B** (4): Investments in vaccine research -+> Creativity -+> Availability of vaccine --> **ASF positive domestic pigs** -+> Investments in vaccine research

12) **B** (4): Investments in vaccine research -+> Creativity -+> Availability of vaccine --> **ASF positive cases in wild boar** -+> Investments in vaccine research

13) **R** (4): **ASF positive domestic pigs** -+> Outbreak personnel (administrative, operational) -+> Hunting activity -+> Humans acting as vectors -+> **ASF positive domestic pigs**

14) **B** (4): Outbreak personnel (administrative, operational) -+> Hunting activity --> Wild boar population -+> Sampling of wild boar -+> Outbreak personnel (administrative, operational)

15) **R** (4): Outbreak personnel (administrative, operational) -+> Hunting activity -+> Sampling of wild boar -+> **ASF positive cases in wild boar** -+> Outbreak personnel (administrative, operational)

16) **R** (4): Outbreak personnel (administrative, operational) -+> Hunting activity -+> Humans acting as vectors -+> **ASF positive cases in wild boar** -+> Outbreak personnel (administrative, operational)

17) **B** (5): **ASF positive domestic pigs** -+> Outbreak personnel (administrative, operational) -+> Education / Awareness raising -+> Qualification and awareness --> Humans acting as vectors -+> **ASF positive domestic pigs**

18) **B** (5): Education / Awareness raising -+> Qualification and awareness --> Humans acting as vectors -+> **ASF positive cases in wild boar** -+> Outbreak personnel (administrative, operational) -+> Education / Awareness raising

19) **B** (5): Outbreak personnel (administrative, operational) -+> Hunting activity --> Wild boar population -+> Sampling of wild boar -+> **ASF positive cases in wild boar** -+> Outbreak personnel (administrative, operational)

20) **B** (6): Size of restriction zones I and II --> Number of domestic pig holdings -+> Sampling of domestic pigs -+> Outbreak personnel (administrative, operational) -+> Sampling of wild boar -+> **ASF positive cases in wild boar** -+> Size of restriction zones I and II

21) **R** (6): Collaboration of research institutions and zoos -+> Investments in vaccine research -+> Creativity -+> Availability of vaccine --> **ASF positive domestic pigs** --> Number of pig holdings in zoos -+> Collaboration of research institutions and zoos

22) **B** (6): Number of domestic pig holdings -+> Sampling of domestic pigs -+> Outbreak personnel (administrative, operational) -+> Hunting activity -+> Humans acting as vectors -+> **ASF positive domestic pigs** --> Number of domestic pig holdings

23) **B** (6): Outbreak personnel (administrative, operational) -+> Hunting activity -+> Humans acting as vectors -+> **ASF positive cases in wild boar** --> Wild boar population -+> Sampling of wild boar -+> Outbreak personnel (administrative, operational)

24) **B** (7): Size of restriction zones I and II --> Number of domestic pig holdings -+> Sampling of domestic pigs -+> **ASF positive domestic pigs** -+> Outbreak personnel (administrative, operational) -+> Sampling of wild boar -+> **ASF positive cases in wild boar** -+> Size of restriction zones I and II

25) **B** (7): Size of restriction zones I and II --> Number of domestic pig holdings -+> Sampling of domestic pigs -+> Outbreak personnel (administrative, operational) -+> Hunting activity -+> Sampling of wild boar -+> **ASF positive cases in wild boar** -+> Size of restriction zones I and II

26) **B** (7): Size of restriction zones I and II --> Number of domestic pig holdings -+> Sampling of domestic pigs -+> Outbreak personnel (administrative, operational) -+> Hunting activity -+> Humans acting as vectors -+> **ASF positive cases in wild boar** -+> Size of restriction zones I and II

27) **R** (7): Size of restriction zones I and II --> Number of pig holdings in zoos -+> Collaboration of research institutions and zoos -+> Investments in vaccine research -+> Creativity -+> Availability of vaccine --> **ASF positive cases in wild boar** -+> Size of restriction zones I and II

28) **B** (7): Investments in vaccine research -+> Creativity -+> Availability of vaccine --> **ASF positive domestic pigs** -+> Outbreak personnel (administrative, operational) -+> Sampling of wild boar -+> **ASF positive cases in wild boar** -+> Investments in vaccine research

29) **B** (7): Investments in vaccine research -+> Creativity -+> Availability of vaccine --> **ASF positive cases in wild boar** -+> Outbreak personnel (administrative, operational) -+> Sampling of domestic pigs -+> **ASF positive domestic pigs** -+> Investments in vaccine research

30) **R** (7): Number of domestic pig holdings -+> Sampling of domestic pigs -+> Outbreak personnel (administrative, operational) -+> Education / Awareness raising -+> Qualification and awareness --> Humans acting as vectors -+> **ASF positive domestic pigs** --> Number of domestic pig holdings

31) **R** (7): Number of domestic pig holdings --> Pressure on agriculture and food industry -+> Awareness of private investors -+> Money -+> Outbreak personnel (administrative, operational) -+> Sampling of domestic pigs -+> **ASF positive domestic pigs** --> Number of domestic pig holdings

32) **R** (7): **ASF positive domestic pigs** --> Number of pig holdings in zoos --> Awareness of public/media -+> Awareness of politics -+> Money -+> Outbreak personnel (administrative, operational) -+> Sampling of domestic pigs -+> **ASF positive domestic pigs**

33) **R** (7): **ASF positive domestic pigs** --> Number of pig holdings in zoos --> Awareness of public/media -+> Awareness of private investors -+> Money -+> Outbreak personnel (administrative, operational) -+> Sampling of domestic pigs -+> **ASF positive domestic pigs**

34) **R** (7): Education / Awareness raising -+> Qualification and awareness --> Humans acting as vectors -+> **ASF positive cases in wild boar** --> Wild boar population -+> Sampling of wild boar -+> Outbreak personnel (administrative, operational) -+> Education / Awareness raising

35) **R** (8): Size of restriction zones I and II --> Number of domestic pig holdings -+> Sampling of domestic pigs -+> **ASF positive domestic pigs** -+> Investments in vaccine research -+> Creativity -+> Availability of vaccine --> **ASF positive cases in wild boar** -+> Size of restriction zones I and II

36) **B** (8): Size of restriction zones I and II --> Number of domestic pig holdings -+> Sampling of domestic pigs -+> **ASF positive domestic pigs** -+> Outbreak personnel (administrative, operational) -+> Hunting activity -+> Sampling of wild boar -+> **ASF positive cases in wild boar** -+> Size of restriction zones I and II

37) **B** (8): Size of restriction zones I and II --> Number of domestic pig holdings -+> Sampling of domestic pigs -+> **ASF positive domestic pigs** -+> Outbreak personnel (administrative, operational) -+> Hunting activity -+> Humans acting as vectors -+> **ASF positive cases in wild boar** -+> Size of restriction zones I and II

38) **R** (8): Size of restriction zones I and II --> Number of domestic pig holdings -+> Sampling of domestic pigs -+> Outbreak personnel (administrative, operational) -+> Education / Awareness raising -+> Qualification and awareness --> Humans acting as vectors -+> **ASF positive cases in wild boar** -+> Size of restriction zones I and II

39) **R** (8): Size of restriction zones I and II --> Number of domestic pig holdings -+> Sampling of domestic pigs -+> Outbreak personnel (administrative, operational) -+> Hunting activity --> Wild boar population -+> Sampling of wild boar -+> **ASF positive cases in wild boar** -+> Size of restriction zones I and II

40) **R** (8): Size of restriction zones I and II --> Number of domestic pig holdings --> Pressure on agriculture and food industry -+> Awareness of private investors -+> Money -+> Outbreak personnel (administrative, operational) -+> Sampling of wild boar -+> **ASF positive cases in wild boar** -+> Size of restriction zones I and II

41) **R** (8): Size of restriction zones I and II --> Number of pig holdings in zoos --> Awareness of public/media -+> Awareness of politics -+> Money -+> Outbreak personnel (administrative, operational) -+> Sampling of wild boar -+> **ASF positive cases in wild boar** -+> Size of restriction zones I and II

42) **R** (8): Size of restriction zones I and II --> Number of pig holdings in zoos --> Awareness of public/media -+> Awareness of private investors -+> Money -+> Outbreak personnel (administrative, operational) -+> Sampling of wild boar -+> **ASF positive cases in wild boar** -+> Size of restriction zones I and II

43) **B** (8): Investments in vaccine research -+> Creativity -+> Availability of vaccine --> **ASF positive domestic pigs** --> Number of domestic pig holdings --> Pressure on agriculture and food industry -+> Awareness of private investors -+> Money -+> Investments in vaccine research

44) **B** (8): Investments in vaccine research -+> Creativity -+> Availability of vaccine --> **ASF positive domestic pigs** --> Number of pig holdings in zoos --> Awareness of public/media -+> Awareness of politics -+> Money -+> Investments in vaccine research

45) **B** (8): Investments in vaccine research -+> Creativity -+> Availability of vaccine --> **ASF positive domestic pigs** --> Number of pig holdings in zoos --> Awareness of public/media -+> Awareness of private investors -+> Money -+> Investments in vaccine research

46) **B** (8): Investments in vaccine research -+> Creativity -+> Availability of vaccine --> **ASF positive domestic pigs** -+> Outbreak personnel (administrative, operational) -+> Hunting activity -+> Sampling of wild boar -+> **ASF positive cases in wild boar** -+> Investments in vaccine research

47) **B** (8): Investments in vaccine research -+> Creativity -+> Availability of vaccine --> **ASF positive domestic pigs** -+> Outbreak personnel (administrative, operational) -+> Hunting activity -+>

Humans acting as vectors -+> ASF positive cases in wild boar -+> Investments in vaccine research

48) **B** (8): Investments in vaccine research -+> Creativity -+> Availability of vaccine --> ASF positive cases in wild boar -+> Outbreak personnel (administrative, operational) -+> Hunting activity -+> Humans acting as vectors -+> **ASF positive domestic pigs** -+> Investments in vaccine research

49) **R** (8): Number of domestic pig holdings --> Pressure on agriculture and food industry -+> Awareness of private investors -+> Money -+> Motivation -+> Outbreak personnel (administrative, operational) -+> Sampling of domestic pigs -+> **ASF positive domestic pigs** --> Number of domestic pig holdings

50) **R** (8): Number of domestic pig holdings --> Pressure on agriculture and food industry -+> Awareness of private investors -+> Money -+> Outbreak personnel (administrative, operational) -+> Hunting activity -+> Humans acting as vectors -+> **ASF positive domestic pigs** --> Number of domestic pig holdings

51) **R** (8): **ASF positive domestic pigs** --> Number of pig holdings in zoos --> Awareness of public/media -+> Awareness of politics -+> Money -+> Motivation -+> Outbreak personnel (administrative, operational) -+> Sampling of domestic pigs -+> **ASF positive domestic pigs**

52) **R** (8): **ASF positive domestic pigs** --> Number of pig holdings in zoos --> Awareness of public/media -+> Awareness of politics -+> Money -+> Outbreak personnel (administrative, operational) -+> Hunting activity -+> Humans acting as vectors -+> **ASF positive domestic pigs**

53) **R** (8): **ASF positive domestic pigs** --> Number of pig holdings in zoos --> Awareness of public/media -+> Awareness of private investors -+> Money -+> Motivation -+> Outbreak personnel (administrative, operational) -+> Sampling of domestic pigs -+> **ASF positive domestic pigs**

54) **R** (8): **ASF positive domestic pigs** --> Number of pig holdings in zoos --> Awareness of public/media -+> Awareness of private investors -+> Money -+> Outbreak personnel (administrative, operational) -+> Hunting activity -+> Humans acting as vectors -+> **ASF positive domestic pigs**

55) **R** (9): Size of restriction zones I and II --> Number of domestic pig holdings -+> Sampling of domestic pigs -+> **ASF positive domestic pigs** -+> Outbreak personnel (administrative, operational) -+> Education / Awareness raising -+> Qualification and awareness --> Humans acting as vectors -+> ASF positive cases in wild boar -+> Size of restriction zones I and II

56) **R** (9): Size of restriction zones I and II --> Number of domestic pig holdings -+> Sampling of domestic pigs -+> **ASF positive domestic pigs** -+> Outbreak personnel (administrative, operational) -+> Hunting activity --> Wild boar population -+> Sampling of wild boar -+> ASF positive cases in wild boar -+> Size of restriction zones I and II

57) **B** (9): Size of restriction zones I and II --> Number of domestic pig holdings --> Pressure on agriculture and food industry -+> Awareness of private investors -+> Money -+> Investments in vaccine research -+> Creativity -+> Availability of vaccine --> ASF positive cases in wild boar -+> Size of restriction zones I and II

58) **R** (9): Size of restriction zones I and II --> Number of domestic pig holdings --> Pressure on agriculture and food industry -+> Awareness of private investors -+> Money -+> Motivation -+> Outbreak personnel (administrative, operational) -+> Sampling of wild boar -+> ASF positive cases in wild boar -+> Size of restriction zones I and II

59) **R** (9): Size of restriction zones I and II --> Number of domestic pig holdings --> Pressure on agriculture and food industry -+> Awareness of private investors -+> Money -+> Outbreak

personnel (administrative, operational) -+> Hunting activity -+> Sampling of wild boar -+> ASF positive cases in wild boar -+> Size of restriction zones I and II

60) **R** (9): Size of restriction zones I and II --> Number of domestic pig holdings --> Pressure on agriculture and food industry -+> Awareness of private investors -+> Money -+> Outbreak personnel (administrative, operational) -+> Hunting activity -+> Humans acting as vectors -+> ASF positive cases in wild boar -+> Size of restriction zones I and II

61) **B** (9): Size of restriction zones I and II --> Number of pig holdings in zoos --> Awareness of public/media -+> Awareness of politics -+> Money -+> Investments in vaccine research -+> Creativity -+> Availability of vaccine --> ASF positive cases in wild boar -+> Size of restriction zones I and II

62) **R** (9): Size of restriction zones I and II --> Number of pig holdings in zoos --> Awareness of public/media -+> Awareness of politics -+> Money -+> Motivation -+> Outbreak personnel (administrative, operational) -+> Sampling of wild boar -+> ASF positive cases in wild boar -+> Size of restriction zones I and II

63) **R** (9): Size of restriction zones I and II --> Number of pig holdings in zoos --> Awareness of public/media -+> Awareness of politics -+> Money -+> Outbreak personnel (administrative, operational) -+> Hunting activity -+> Sampling of wild boar -+> ASF positive cases in wild boar -+> Size of restriction zones I and II

64) **R** (9): Size of restriction zones I and II --> Number of pig holdings in zoos --> Awareness of public/media -+> Awareness of politics -+> Money -+> Outbreak personnel (administrative, operational) -+> Hunting activity -+> Humans acting as vectors -+> ASF positive cases in wild boar -+> Size of restriction zones I and II

65) **B** (9): Size of restriction zones I and II --> Number of pig holdings in zoos --> Awareness of public/media -+> Awareness of private investors -+> Money -+> Investments in vaccine research -+> Creativity -+> Availability of vaccine --> ASF positive cases in wild boar -+> Size of restriction zones I and II

66) **R** (9): Size of restriction zones I and II --> Number of pig holdings in zoos --> Awareness of public/media -+> Awareness of private investors -+> Money -+> Motivation -+> Outbreak personnel (administrative, operational) -+> Sampling of wild boar -+> ASF positive cases in wild boar -+> Size of restriction zones I and II

67) **R** (9): Size of restriction zones I and II --> Number of pig holdings in zoos --> Awareness of public/media -+> Awareness of private investors -+> Money -+> Outbreak personnel (administrative, operational) -+> Hunting activity -+> Sampling of wild boar -+> ASF positive cases in wild boar -+> Size of restriction zones I and II

68) **R** (9): Size of restriction zones I and II --> Number of pig holdings in zoos --> Awareness of public/media -+> Awareness of private investors -+> Money -+> Outbreak personnel (administrative, operational) -+> Hunting activity -+> Humans acting as vectors -+> ASF positive cases in wild boar -+> Size of restriction zones I and II

69) **R** (9): Collaboration of research institutions and zoos -+> Investments in vaccine research -+> Creativity -+> Availability of vaccine --> ASF positive cases in wild boar -+> Outbreak personnel (administrative, operational) -+> Sampling of domestic pigs -+> **ASF positive domestic pigs** --> Number of pig holdings in zoos -+> Collaboration of research institutions and zoos

70) **R** (9): Investments in vaccine research -+> Creativity -+> Availability of vaccine --> **ASF positive domestic pigs** --> Number of domestic pig holdings -+> Sampling of domestic pigs -+> Outbreak personnel (administrative, operational) -+> Sampling of wild boar -+> ASF positive cases in wild boar -+> Investments in vaccine research

71) **R** (9): Investments in vaccine research -+> Creativity -+> Availability of vaccine --> **ASF positive domestic pigs** -+> Outbreak personnel (administrative, operational) -+> Education / Awareness raising -+> Qualification and awareness --> Humans acting as vectors -+> **ASF positive cases in wild boar** -+> Investments in vaccine research

72) **R** (9): Investments in vaccine research -+> Creativity -+> Availability of vaccine --> **ASF positive domestic pigs** -+> Outbreak personnel (administrative, operational) -+> Hunting activity --> Wild boar population -+> Sampling of wild boar -+> **ASF positive cases in wild boar** -+> Investments in vaccine research

73) **R** (9): Investments in vaccine research -+> Creativity -+> Availability of vaccine --> **ASF positive cases in wild boar** -+> Outbreak personnel (administrative, operational) -+> Education / Awareness raising -+> Qualification and awareness --> Humans acting as vectors -+> **ASF positive domestic pigs** -+> Investments in vaccine research

74) **R** (9): Investments in vaccine research -+> Creativity -+> Availability of vaccine --> **ASF positive cases in wild boar** --> Wild boar population -+> Sampling of wild boar -+> Outbreak personnel (administrative, operational) -+> Sampling of domestic pigs -+> **ASF positive domestic pigs** -+> Investments in vaccine research

75) **R** (9): Number of domestic pig holdings --> Pressure on agriculture and food industry -+> Awareness of private investors -+> Money -+> Motivation -+> Outbreak personnel (administrative, operational) -+> Hunting activity -+> Humans acting as vectors -+> **ASF positive domestic pigs** --> Number of domestic pig holdings

76) **B** (9): Number of domestic pig holdings --> Pressure on agriculture and food industry -+> Awareness of private investors -+> Money -+> Outbreak personnel (administrative, operational) -+> Education / Awareness raising -+> Qualification and awareness --> Humans acting as vectors -+> **ASF positive domestic pigs** --> Number of domestic pig holdings

77) **R** (9): **ASF positive domestic pigs** --> Number of pig holdings in zoos --> Awareness of public/media -+> Awareness of politics -+> Money -+> Motivation -+> Outbreak personnel (administrative, operational) -+> Hunting activity -+> Humans acting as vectors -+> **ASF positive domestic pigs**

78) **B** (9): **ASF positive domestic pigs** --> Number of pig holdings in zoos --> Awareness of public/media -+> Awareness of politics -+> Money -+> Outbreak personnel (administrative, operational) -+> Education / Awareness raising -+> Qualification and awareness --> Humans acting as vectors -+> **ASF positive domestic pigs**

79) **R** (9): **ASF positive domestic pigs** --> Number of pig holdings in zoos --> Awareness of public/media -+> Awareness of private investors -+> Money -+> Motivation -+> Outbreak personnel (administrative, operational) -+> Hunting activity -+> Humans acting as vectors -+> **ASF positive domestic pigs**

80) **B** (9): **ASF positive domestic pigs** --> Number of pig holdings in zoos --> Awareness of public/media -+> Awareness of private investors -+> Money -+> Outbreak personnel (administrative, operational) -+> Education / Awareness raising -+> Qualification and awareness --> Humans acting as vectors -+> **ASF positive domestic pigs**

81) **B** (10): Size of restriction zones I and II --> Number of domestic pig holdings -+> Sampling of domestic pigs -+> **ASF positive domestic pigs** --> Number of pig holdings in zoos -+> Collaboration of research institutions and zoos -+> Investments in vaccine research -+> Creativity -+> Availability of vaccine --> **ASF positive cases in wild boar** -+> Size of restriction zones I and II

82) **R** (10): Size of restriction zones I and II --> Number of domestic pig holdings --> Pressure on agriculture and food industry -+> Awareness of private investors -+> Money -+> Motivation -+> Outbreak personnel (administrative, operational) -+> Hunting activity -+> Sampling of wild boar -+> **ASF positive cases in wild boar** -+> Size of restriction zones I and II

83) **R** (10): Size of restriction zones I and II --> Number of domestic pig holdings --> Pressure on agriculture and food industry -+> Awareness of private investors -+> Money -+> Motivation -+> Outbreak personnel (administrative, operational) -+> Hunting activity -+> Humans acting as vectors -+> **ASF positive cases in wild boar** -+> Size of restriction zones I and II

84) **B** (10): Size of restriction zones I and II --> Number of domestic pig holdings --> Pressure on agriculture and food industry -+> Awareness of private investors -+> Money -+> Outbreak personnel (administrative, operational) -+> Education / Awareness raising -+> Qualification and awareness --> Humans acting as vectors -+> **ASF positive cases in wild boar** -+> Size of restriction zones I and II

85) **B** (10): Size of restriction zones I and II --> Number of domestic pig holdings --> Pressure on agriculture and food industry -+> Awareness of private investors -+> Money -+> Outbreak personnel (administrative, operational) -+> Hunting activity --> Wild boar population -+> Sampling of wild boar -+> **ASF positive cases in wild boar** -+> Size of restriction zones I and II

86) **R** (10): Size of restriction zones I and II --> Number of pig holdings in zoos -+> Collaboration of research institutions and zoos -+> Investments in vaccine research -+> Creativity -+> Availability of vaccine --> **ASF positive domestic pigs** -+> Outbreak personnel (administrative, operational) -+> Sampling of wild boar -+> **ASF positive cases in wild boar** -+> Size of restriction zones I and II

87) **R** (10): Size of restriction zones I and II --> Number of pig holdings in zoos --> Awareness of public/media -+> Awareness of politics -+> Money -+> Motivation -+> Outbreak personnel (administrative, operational) -+> Hunting activity -+> Sampling of wild boar -+> **ASF positive cases in wild boar** -+> Size of restriction zones I and II

88) **R** (10): Size of restriction zones I and II --> Number of pig holdings in zoos --> Awareness of public/media -+> Awareness of politics -+> Money -+> Motivation -+> Outbreak personnel (administrative, operational) -+> Hunting activity -+> Humans acting as vectors -+> **ASF positive cases in wild boar** -+> Size of restriction zones I and II

89) **B** (10): Size of restriction zones I and II --> Number of pig holdings in zoos --> Awareness of public/media -+> Awareness of politics -+> Money -+> Outbreak personnel (administrative, operational) -+> Education / Awareness raising -+> Qualification and awareness --> Humans acting as vectors -+> **ASF positive cases in wild boar** -+> Size of restriction zones I and II

90) **B** (10): Size of restriction zones I and II --> Number of pig holdings in zoos --> Awareness of public/media -+> Awareness of politics -+> Money -+> Outbreak personnel (administrative, operational) -+> Hunting activity --> Wild boar population -+> Sampling of wild boar -+> **ASF positive cases in wild boar** -+> Size of restriction zones I and II

91) **R** (10): Size of restriction zones I and II --> Number of pig holdings in zoos --> Awareness of public/media -+> Awareness of private investors -+> Money -+> Motivation -+> Outbreak personnel (administrative, operational) -+> Hunting activity -+> Sampling of wild boar -+> **ASF positive cases in wild boar** -+> Size of restriction zones I and II

92) **R** (10): Size of restriction zones I and II --> Number of pig holdings in zoos --> Awareness of public/media -+> Awareness of private investors -+> Money -+> Motivation -+> Outbreak personnel (administrative, operational) -+> Hunting activity -+> Humans acting as vectors -+> **ASF positive cases in wild boar** -+> Size of restriction zones I and II

93) **B** (10): Size of restriction zones I and II --> Number of pig holdings in zoos --> Awareness of public/media -+> Awareness of private investors -+> Money -+> Outbreak personnel (administrative, operational) -+> Education / Awareness raising -+> Qualification and awareness --> Humans acting as vectors -+> **ASF positive cases in wild boar** -+> Size of restriction zones I and II

94) **B** (10): Size of restriction zones I and II --> Number of pig holdings in zoos --> Awareness of public/media -+> Awareness of private investors -+> Money -+> Outbreak personnel (administrative, operational) -+> Hunting activity --> Wild boar population -+> Sampling of wild boar -+> **ASF positive cases in wild boar** -+> Size of restriction zones I and II

95) **R** (10): Collaboration of research institutions and zoos -+> Investments in vaccine research -+> Creativity -+> Availability of vaccine --> **ASF positive cases in wild boar** -+> Outbreak personnel (administrative, operational) -+> Hunting activity -+> Humans acting as vectors -+> **ASF positive domestic pigs** --> Number of pig holdings in zoos -+> Collaboration of research institutions and zoos

96) **R** (10): Investments in vaccine research -+> Creativity -+> Availability of vaccine --> **ASF positive domestic pigs** --> Number of domestic pig holdings -+> Sampling of domestic pigs -+> Outbreak personnel (administrative, operational) -+> Hunting activity -+> Sampling of wild boar -+> **ASF positive cases in wild boar** -+> Investments in vaccine research

97) **R** (10): Investments in vaccine research -+> Creativity -+> Availability of vaccine --> **ASF positive domestic pigs** --> Number of domestic pig holdings -+> Sampling of domestic pigs -+> Outbreak personnel (administrative, operational) -+> Hunting activity -+> Humans acting as vectors -+> **ASF positive cases in wild boar** -+> Investments in vaccine research

98) **R** (10): Investments in vaccine research -+> Creativity -+> Availability of vaccine --> **ASF positive cases in wild boar** --> Wild boar population -+> Sampling of wild boar -+> Outbreak personnel (administrative, operational) -+> Hunting activity -+> Humans acting as vectors -+> **ASF positive domestic pigs** -+> Investments in vaccine research

99) **B** (10): Number of domestic pig holdings --> Pressure on agriculture and food industry -+> Awareness of private investors -+> Money -+> Motivation -+> Outbreak personnel (administrative, operational) -+> Education / Awareness raising -+> Qualification and awareness --> Humans acting as vectors -+> **ASF positive domestic pigs** --> Number of domestic pig holdings

100) **B** (10): **ASF positive domestic pigs** --> Number of pig holdings in zoos --> Awareness of public/media -+> Awareness of politics -+> Money -+> Motivation -+> Outbreak personnel (administrative, operational) -+> Education / Awareness raising -+> Qualification and awareness --> Humans acting as vectors -+> **ASF positive domestic pigs**

101) **B** (10): **ASF positive domestic pigs** --> Number of pig holdings in zoos --> Awareness of public/media -+> Awareness of private investors -+> Money -+> Motivation -+> Outbreak personnel (administrative, operational) -+> Education / Awareness raising -+> Qualification and awareness --> Humans acting as vectors -+> **ASF positive domestic pigs**

102) **B** (11): Size of restriction zones I and II --> Number of domestic pig holdings -+> Sampling of domestic pigs -+> **ASF positive domestic pigs** --> Number of pig holdings in zoos --> Awareness of public/media -+> Awareness of politics -+> Money -+> Outbreak personnel (administrative, operational) -+> Sampling of wild boar -+> **ASF positive cases in wild boar** -+> Size of restriction zones I and II

103) **B** (11): Size of restriction zones I and II --> Number of domestic pig holdings -+> Sampling of domestic pigs -+> **ASF positive domestic pigs** --> Number of pig holdings in zoos --> Awareness

of public/media -+> Awareness of private investors -+> Money -+> Outbreak personnel (administrative, operational) -+> Sampling of wild boar -+> **ASF positive cases in wild boar** -+> Size of restriction zones I and II

104) **R** (11): Size of restriction zones I and II --> Number of domestic pig holdings -+> Sampling of domestic pigs -+> Outbreak personnel (administrative, operational) -+> Hunting activity -+> Humans acting as vectors -+> **ASF positive domestic pigs** -+> Investments in vaccine research -+> Creativity -+> Availability of vaccine --> **ASF positive cases in wild boar** -+> Size of restriction zones I and II

105) **B** (11): Size of restriction zones I and II --> Number of domestic pig holdings --> Pressure on agriculture and food industry -+> Awareness of private investors -+> Money -+> Motivation -+> Outbreak personnel (administrative, operational) -+> Education / Awareness raising -+> Qualification and awareness --> Humans acting as vectors -+> **ASF positive cases in wild boar** -+> Size of restriction zones I and II

106) **B** (11): Size of restriction zones I and II --> Number of domestic pig holdings --> Pressure on agriculture and food industry -+> Awareness of private investors -+> Money -+> Motivation -+> Outbreak personnel (administrative, operational) -+> Hunting activity --> Wild boar population -+> Sampling of wild boar -+> **ASF positive cases in wild boar** -+> Size of restriction zones I and II

107) **R** (11): Size of restriction zones I and II --> Number of pig holdings in zoos -+> Collaboration of research institutions and zoos -+> Investments in vaccine research -+> Creativity -+> Availability of vaccine --> **ASF positive domestic pigs** -+> Outbreak personnel (administrative, operational) -+> Hunting activity -+> Sampling of wild boar -+> **ASF positive cases in wild boar** -+> Size of restriction zones I and II

108) **R** (11): Size of restriction zones I and II --> Number of pig holdings in zoos -+> Collaboration of research institutions and zoos -+> Investments in vaccine research -+> Creativity -+> Availability of vaccine --> **ASF positive domestic pigs** -+> Outbreak personnel (administrative, operational) -+> Hunting activity -+> Humans acting as vectors -+> **ASF positive cases in wild boar** -+> Size of restriction zones I and II

109) **B** (11): Size of restriction zones I and II --> Number of pig holdings in zoos --> Awareness of public/media -+> Awareness of politics -+> Money -+> Motivation -+> Outbreak personnel (administrative, operational) -+> Education / Awareness raising -+> Qualification and awareness --> Humans acting as vectors -+> **ASF positive cases in wild boar** -+> Size of restriction zones I and II

110) **B** (11): Size of restriction zones I and II --> Number of pig holdings in zoos --> Awareness of public/media -+> Awareness of politics -+> Money -+> Motivation -+> Outbreak personnel (administrative, operational) -+> Hunting activity --> Wild boar population -+> Sampling of wild boar -+> **ASF positive cases in wild boar** -+> Size of restriction zones I and II

111) **B** (11): Size of restriction zones I and II --> Number of pig holdings in zoos --> Awareness of public/media -+> Awareness of private investors -+> Money -+> Motivation -+> Outbreak personnel (administrative, operational) -+> Education / Awareness raising -+> Qualification and awareness --> Humans acting as vectors -+> **ASF positive cases in wild boar** -+> Size of restriction zones I and II

112) **B** (11): Size of restriction zones I and II --> Number of pig holdings in zoos --> Awareness of public/media -+> Awareness of private investors -+> Money -+> Motivation -+> Outbreak personnel (administrative, operational) -+> Hunting activity --> Wild boar population -+> Sampling of wild boar -+> **ASF positive cases in wild boar** -+> Size of restriction zones I and II

113) **B** (11): Collaboration of research institutions and zoos -+> Investments in vaccine research -+> Creativity -+> Availability of vaccine --> **ASF positive cases in wild boar** -+> Outbreak personnel (administrative, operational) -+> Education / Awareness raising -+> Qualification and awareness --> Humans acting as vectors -+> **ASF positive domestic pigs** --> Number of pig holdings in zoos -+> Collaboration of research institutions and zoos

114) **B** (11): Collaboration of research institutions and zoos -+> Investments in vaccine research -+> Creativity -+> Availability of vaccine --> **ASF positive cases in wild boar** --> Wild boar population -+> Sampling of wild boar -+> Outbreak personnel (administrative, operational) -+> Sampling of domestic pigs -+> **ASF positive domestic pigs** --> Number of pig holdings in zoos -+> Collaboration of research institutions and zoos

115) **B** (11): Investments in vaccine research -+> Creativity -+> Availability of vaccine --> **ASF positive domestic pigs** --> Number of domestic pig holdings -+> Sampling of domestic pigs -+> Outbreak personnel (administrative, operational) -+> Education / Awareness raising -+> Qualification and awareness --> Humans acting as vectors -+> **ASF positive cases in wild boar** -+> Investments in vaccine research

116) **B** (11): Investments in vaccine research -+> Creativity -+> Availability of vaccine --> **ASF positive domestic pigs** --> Number of domestic pig holdings -+> Sampling of domestic pigs -+> Outbreak personnel (administrative, operational) -+> Hunting activity --> Wild boar population -+> Sampling of wild boar -+> **ASF positive cases in wild boar** -+> Investments in vaccine research

117) **B** (11): Investments in vaccine research -+> Creativity -+> Availability of vaccine --> **ASF positive domestic pigs** --> Number of domestic pig holdings --> Pressure on agriculture and food industry -+> Awareness of private investors -+> Money -+> Outbreak personnel (administrative, operational) -+> Sampling of wild boar -+> **ASF positive cases in wild boar** -+> Investments in vaccine research

118) **B** (11): Investments in vaccine research -+> Creativity -+> Availability of vaccine --> **ASF positive domestic pigs** --> Number of pig holdings in zoos --> Awareness of public/media -+> Awareness of politics -+> Money -+> Outbreak personnel (administrative, operational) -+> Sampling of wild boar -+> **ASF positive cases in wild boar** -+> Investments in vaccine research

119) **B** (11): Investments in vaccine research -+> Creativity -+> Availability of vaccine --> **ASF positive domestic pigs** --> Number of pig holdings in zoos --> Awareness of public/media -+> Awareness of private investors -+> Money -+> Outbreak personnel (administrative, operational) -+> Sampling of wild boar -+> **ASF positive cases in wild boar** -+> Investments in vaccine research

120) **B** (11): Investments in vaccine research -+> Creativity -+> Availability of vaccine --> **ASF positive cases in wild boar** -+> Outbreak personnel (administrative, operational) -+> Sampling of domestic pigs -+> **ASF positive domestic pigs** --> Number of domestic pig holdings --> Pressure on agriculture and food industry -+> Awareness of private investors -+> Money -+> Investments in vaccine research

121) **B** (11): Investments in vaccine research -+> Creativity -+> Availability of vaccine --> **ASF positive cases in wild boar** -+> Outbreak personnel (administrative, operational) -+> Sampling of domestic pigs -+> **ASF positive domestic pigs** --> Number of pig holdings in zoos --> Awareness of public/media -+> Awareness of politics -+> Money -+> Investments in vaccine research

122) **B** (11): Investments in vaccine research -+> Creativity -+> Availability of vaccine --> **ASF positive cases in wild boar** -+> Outbreak personnel (administrative, operational) -+> Sampling of domestic pigs -+> **ASF positive domestic pigs** --> Number of pig holdings in zoos --> Awareness of public/media -+> Awareness of private investors -+> Money -+> Investments in vaccine research

123) **B** (11): Investments in vaccine research -+> Creativity -+> Availability of vaccine --> **ASF positive cases in wild boar** --> Wild boar population -+> Sampling of wild boar -+> Outbreak personnel (administrative, operational) -+> Education / Awareness raising -+> Qualification and awareness --> Humans acting as vectors -+> **ASF positive domestic pigs** -+> Investments in vaccine research

124) **R** (12): Size of restriction zones I and II --> Number of domestic pig holdings -+> Sampling of domestic pigs -+> **ASF positive domestic pigs** --> Number of pig holdings in zoos --> Awareness of public/media -+> Awareness of politics -+> Money -+> Investments in vaccine research -+> Creativity -+> Availability of vaccine --> **ASF positive cases in wild boar** -+> Size of restriction zones I and II

125) **B** (12): Size of restriction zones I and II --> Number of domestic pig holdings -+> Sampling of domestic pigs -+> **ASF positive domestic pigs** --> Number of pig holdings in zoos --> Awareness of public/media -+> Awareness of politics -+> Money -+> Motivation -+> Outbreak personnel (administrative, operational) -+> Sampling of wild boar -+> **ASF positive cases in wild boar** -+> Size of restriction zones I and II

126) **B** (12): Size of restriction zones I and II --> Number of domestic pig holdings -+> Sampling of domestic pigs -+> **ASF positive domestic pigs** --> Number of pig holdings in zoos --> Awareness of public/media -+> Awareness of politics -+> Money -+> Outbreak personnel (administrative, operational) -+> Hunting activity -+> Sampling of wild boar -+> **ASF positive cases in wild boar** -+> Size of restriction zones I and II

127) **B** (12): Size of restriction zones I and II --> Number of domestic pig holdings -+> Sampling of domestic pigs -+> **ASF positive domestic pigs** --> Number of pig holdings in zoos --> Awareness of public/media -+> Awareness of politics -+> Money -+> Outbreak personnel (administrative, operational) -+> Hunting activity -+> Humans acting as vectors -+> **ASF positive cases in wild boar** -+> Size of restriction zones I and II

128) **R** (12): Size of restriction zones I and II --> Number of domestic pig holdings -+> Sampling of domestic pigs -+> **ASF positive domestic pigs** --> Number of pig holdings in zoos --> Awareness of public/media -+> Awareness of private investors -+> Money -+> Investments in vaccine research -+> Creativity -+> Availability of vaccine --> **ASF positive cases in wild boar** -+> Size of restriction zones I and II

129) **B** (12): Size of restriction zones I and II --> Number of domestic pig holdings -+> Sampling of domestic pigs -+> **ASF positive domestic pigs** --> Number of pig holdings in zoos --> Awareness of public/media -+> Awareness of private investors -+> Money -+> Motivation -+> Outbreak personnel (administrative, operational) -+> Sampling of wild boar -+> **ASF positive cases in wild boar** -+> Size of restriction zones I and II

130) **B** (12): Size of restriction zones I and II --> Number of domestic pig holdings -+> Sampling of domestic pigs -+> **ASF positive domestic pigs** --> Number of pig holdings in zoos --> Awareness of public/media -+> Awareness of private investors -+> Money -+> Outbreak personnel (administrative, operational) -+> Hunting activity -+> Sampling of wild boar -+> **ASF positive cases in wild boar** -+> Size of restriction zones I and II

131) **B** (12): Size of restriction zones I and II --> Number of domestic pig holdings -+> Sampling of domestic pigs -+> **ASF positive domestic pigs** --> Number of pig holdings in zoos --> Awareness of public/media -+> Awareness of private investors -+> Money -+> Outbreak personnel (administrative, operational) -+> Hunting activity -+> Humans acting as vectors -+> **ASF positive cases in wild boar** -+> Size of restriction zones I and II

132) **B** (12): Size of restriction zones I and II --> Number of domestic pig holdings -+> Sampling of domestic pigs -+> Outbreak personnel (administrative, operational) -+> Education / Awareness raising -+> Qualification and awareness --> Humans acting as vectors -+> **ASF positive domestic pigs** -+> Investments in vaccine research -+> Creativity -+> Availability of vaccine --> **ASF positive cases in wild boar** -+> Size of restriction zones I and II

133) **B** (12): Size of restriction zones I and II --> Number of domestic pig holdings --> Pressure on agriculture and food industry -+> Awareness of private investors -+> Money -+> Investments in vaccine research -+> Creativity -+> Availability of vaccine --> **ASF positive domestic pigs** -+> Outbreak personnel (administrative, operational) -+> Sampling of wild boar -+> **ASF positive cases in wild boar** -+> Size of restriction zones I and II

134) **B** (12): Size of restriction zones I and II --> Number of domestic pig holdings --> Pressure on agriculture and food industry -+> Awareness of private investors -+> Money -+> Outbreak personnel (administrative, operational) -+> Sampling of domestic pigs -+> **ASF positive domestic pigs** -+> Investments in vaccine research -+> Creativity -+> Availability of vaccine --> **ASF positive cases in wild boar** -+> Size of restriction zones I and II

135) **B** (12): Size of restriction zones I and II --> Number of pig holdings in zoos -+> Collaboration of research institutions and zoos -+> Investments in vaccine research -+> Creativity -+> Availability of vaccine --> **ASF positive domestic pigs** --> Number of domestic pig holdings -+> Sampling of domestic pigs -+> Outbreak personnel (administrative, operational) -+> Sampling of wild boar -+> **ASF positive cases in wild boar** -+> Size of restriction zones I and II

136) **B** (12): Size of restriction zones I and II --> Number of pig holdings in zoos -+> Collaboration of research institutions and zoos -+> Investments in vaccine research -+> Creativity -+> Availability of vaccine --> **ASF positive domestic pigs** -+> Outbreak personnel (administrative, operational) -+> Education / Awareness raising -+> Qualification and awareness --> Humans acting as vectors -+> **ASF positive cases in wild boar** -+> Size of restriction zones I and II

137) **B** (12): Size of restriction zones I and II --> Number of pig holdings in zoos -+> Collaboration of research institutions and zoos -+> Investments in vaccine research -+> Creativity -+> Availability of vaccine --> **ASF positive domestic pigs** -+> Outbreak personnel (administrative, operational) -+> Hunting activity --> Wild boar population -+> Sampling of wild boar -+> **ASF positive cases in wild boar** -+> Size of restriction zones I and II

138) **B** (12): Size of restriction zones I and II --> Number of pig holdings in zoos --> Awareness of public/media -+> Awareness of politics -+> Money -+> Investments in vaccine research -+> Creativity -+> Availability of vaccine --> **ASF positive domestic pigs** -+> Outbreak personnel (administrative, operational) -+> Sampling of wild boar -+> **ASF positive cases in wild boar** -+> Size of restriction zones I and II

139) **B** (12): Size of restriction zones I and II --> Number of pig holdings in zoos --> Awareness of public/media -+> Awareness of politics -+> Money -+> Outbreak personnel (administrative, operational) -+> Sampling of domestic pigs -+> **ASF positive domestic pigs** -+> Investments in vaccine research -+> Creativity -+> Availability of vaccine --> **ASF positive cases in wild boar** -+> Size of restriction zones I and II

140) **B** (12): Size of restriction zones I and II --> Number of pig holdings in zoos --> Awareness of public/media -+> Awareness of private investors -+> Money -+> Investments in vaccine research -+> Creativity -+> Availability of vaccine --> **ASF positive domestic pigs** -+> Outbreak personnel (administrative, operational) -+> Sampling of wild boar -+> **ASF positive cases in wild boar** -+> Size of restriction zones I and II

141) **B** (12): Size of restriction zones I and II --> Number of pig holdings in zoos --> Awareness of public/media -+> Awareness of private investors -+> Money -+> Outbreak personnel (administrative, operational) -+> Sampling of domestic pigs -+> **ASF positive domestic pigs** -+> Investments in vaccine research -+> Creativity -+> Availability of vaccine --> **ASF positive cases in wild boar** -+> Size of restriction zones I and II

142) **B** (12): Collaboration of research institutions and zoos -+> Investments in vaccine research -+> Creativity -+> Availability of vaccine --> **ASF positive cases in wild boar** --> Wild boar population -+> Sampling of wild boar -+> Outbreak personnel (administrative, operational) -+> Hunting activity -+> Humans acting as vectors -+> **ASF positive domestic pigs** --> Number of pig holdings in zoos -+> Collaboration of research institutions and zoos

143) **B** (12): Investments in vaccine research -+> Creativity -+> Availability of vaccine --> **ASF positive domestic pigs** --> Number of domestic pig holdings --> Pressure on agriculture and food industry -+> Awareness of private investors -+> Money -+> Motivation -+> Outbreak personnel (administrative, operational) -+> Sampling of wild boar -+> **ASF positive cases in wild boar** -+> Investments in vaccine research

144) **B** (12): Investments in vaccine research -+> Creativity -+> Availability of vaccine --> **ASF positive domestic pigs** --> Number of domestic pig holdings --> Pressure on agriculture and food industry -+> Awareness of private investors -+> Money -+> Outbreak personnel (administrative, operational) -+> Hunting activity -+> Sampling of wild boar -+> **ASF positive cases in wild boar** -+> Investments in vaccine research

145) **B** (12): Investments in vaccine research -+> Creativity -+> Availability of vaccine --> **ASF positive domestic pigs** --> Number of domestic pig holdings --> Pressure on agriculture and food industry -+> Awareness of private investors -+> Money -+> Outbreak personnel (administrative, operational) -+> Hunting activity -+> Humans acting as vectors -+> **ASF positive cases in wild boar** -+> Investments in vaccine research

146) **B** (12): Investments in vaccine research -+> Creativity -+> Availability of vaccine --> **ASF positive domestic pigs** --> Number of pig holdings in zoos --> Awareness of public/media -+> Awareness of politics -+> Money -+> Motivation -+> Outbreak personnel (administrative, operational) -+> Sampling of wild boar -+> **ASF positive cases in wild boar** -+> Investments in vaccine research

147) **B** (12): Investments in vaccine research -+> Creativity -+> Availability of vaccine --> **ASF positive domestic pigs** --> Number of pig holdings in zoos --> Awareness of public/media -+> Awareness of politics -+> Money -+> Outbreak personnel (administrative, operational) -+> Hunting activity -+> Sampling of wild boar -+> **ASF positive cases in wild boar** -+> Investments in vaccine research

148) **B** (12): Investments in vaccine research -+> Creativity -+> Availability of vaccine --> **ASF positive domestic pigs** --> Number of pig holdings in zoos --> Awareness of public/media -+> Awareness of politics -+> Money -+> Outbreak personnel (administrative, operational) -+> Hunting activity -+> Humans acting as vectors -+> **ASF positive cases in wild boar** -+> Investments in vaccine research

149) **B** (12): Investments in vaccine research -+> Creativity -+> Availability of vaccine --> **ASF positive domestic pigs** --> Number of pig holdings in zoos --> Awareness of public/media -+> Awareness of private investors -+> Money -+> Motivation -+> Outbreak personnel (administrative, operational) -+> Sampling of wild boar -+> **ASF positive cases in wild boar** -+> Investments in vaccine research

150) **B** (12): Investments in vaccine research -+> Creativity -+> Availability of vaccine --> **ASF positive domestic pigs** --> Number of pig holdings in zoos --> Awareness of public/media -+> Awareness

of private investors -+> Money -+> Outbreak personnel (administrative, operational) -+> Hunting activity -+> Sampling of wild boar -+> **ASF positive cases in wild boar** -+> Investments in vaccine research

151) **B** (12): Investments in vaccine research -+> Creativity -+> Availability of vaccine --> **ASF positive domestic pigs** --> Number of pig holdings in zoos --> Awareness of public/media -+> Awareness of private investors -+> Money -+> Outbreak personnel (administrative, operational) -+> Hunting activity -+> Humans acting as vectors -+> **ASF positive cases in wild boar** -+> Investments in vaccine research

152) **B** (12): Investments in vaccine research -+> Creativity -+> Availability of vaccine --> **ASF positive cases in wild boar** -+> Outbreak personnel (administrative, operational) -+> Hunting activity -+> Humans acting as vectors -+> **ASF positive domestic pigs** --> Number of domestic pig holdings --> Pressure on agriculture and food industry -+> Awareness of private investors -+> Money -+> Investments in vaccine research

153) **B** (12): Investments in vaccine research -+> Creativity -+> Availability of vaccine --> **ASF positive cases in wild boar** -+> Outbreak personnel (administrative, operational) -+> Hunting activity -+> Humans acting as vectors -+> **ASF positive domestic pigs** --> Number of pig holdings in zoos --> Awareness of public/media -+> Awareness of politics -+> Money -+> Investments in vaccine research

154) **B** (12): Investments in vaccine research -+> Creativity -+> Availability of vaccine --> **ASF positive cases in wild boar** -+> Outbreak personnel (administrative, operational) -+> Hunting activity -+> Humans acting as vectors -+> **ASF positive domestic pigs** --> Number of pig holdings in zoos --> Awareness of public/media -+> Awareness of private investors -+> Money -+> Investments in vaccine research

155) **B** (13): Size of restriction zones I and II --> Number of domestic pig holdings -+> Sampling of domestic pigs -+> **ASF positive domestic pigs** --> Number of pig holdings in zoos --> Awareness of public/media -+> Awareness of politics -+> Money -+> Motivation -+> Outbreak personnel (administrative, operational) -+> Hunting activity -+> Sampling of wild boar -+> **ASF positive cases in wild boar** -+> Size of restriction zones I and II

156) **B** (13): Size of restriction zones I and II --> Number of domestic pig holdings -+> Sampling of domestic pigs -+> **ASF positive domestic pigs** --> Number of pig holdings in zoos --> Awareness of public/media -+> Awareness of politics -+> Money -+> Motivation -+> Outbreak personnel (administrative, operational) -+> Hunting activity -+> Humans acting as vectors -+> **ASF positive cases in wild boar** -+> Size of restriction zones I and II

157) **R** (13): Size of restriction zones I and II --> Number of domestic pig holdings -+> Sampling of domestic pigs -+> **ASF positive domestic pigs** --> Number of pig holdings in zoos --> Awareness of public/media -+> Awareness of politics -+> Money -+> Outbreak personnel (administrative, operational) -+> Education / Awareness raising -+> Qualification and awareness --> Humans acting as vectors -+> **ASF positive cases in wild boar** -+> Size of restriction zones I and II

158) **R** (13): Size of restriction zones I and II --> Number of domestic pig holdings -+> Sampling of domestic pigs -+> **ASF positive domestic pigs** --> Number of pig holdings in zoos --> Awareness of public/media -+> Awareness of politics -+> Money -+> Outbreak personnel (administrative, operational) -+> Hunting activity --> Wild boar population -+> Sampling of wild boar -+> **ASF positive cases in wild boar** -+> Size of restriction zones I and II

159) **B** (13): Size of restriction zones I and II --> Number of domestic pig holdings -+> Sampling of domestic pigs -+> **ASF positive domestic pigs** --> Number of pig holdings in zoos --> Awareness of public/media -+> Awareness of private investors -+> Money -+> Motivation -+> Outbreak

personnel (administrative, operational) -+> Hunting activity -+> Sampling of wild boar -+> ASF positive cases in wild boar -+> Size of restriction zones I and II

160) **B** (13): Size of restriction zones I and II --> Number of domestic pig holdings -+> Sampling of domestic pigs -+> **ASF positive domestic pigs** --> Number of pig holdings in zoos --> Awareness of public/media -+> Awareness of private investors -+> Money -+> Motivation -+> Outbreak personnel (administrative, operational) -+> Hunting activity -+> Humans acting as vectors -+> ASF positive cases in wild boar -+> Size of restriction zones I and II

161) **R** (13): Size of restriction zones I and II --> Number of domestic pig holdings -+> Sampling of domestic pigs -+> **ASF positive domestic pigs** --> Number of pig holdings in zoos --> Awareness of public/media -+> Awareness of private investors -+> Money -+> Outbreak personnel (administrative, operational) -+> Education / Awareness raising -+> Qualification and awareness --> Humans acting as vectors -+> ASF positive cases in wild boar -+> Size of restriction zones I and II

162) **R** (13): Size of restriction zones I and II --> Number of domestic pig holdings -+> Sampling of domestic pigs -+> **ASF positive domestic pigs** --> Number of pig holdings in zoos --> Awareness of public/media -+> Awareness of private investors -+> Money -+> Outbreak personnel (administrative, operational) -+> Hunting activity --> Wild boar population -+> Sampling of wild boar -+> ASF positive cases in wild boar -+> Size of restriction zones I and II

163) **B** (13): Size of restriction zones I and II --> Number of domestic pig holdings -+> Sampling of domestic pigs -+> Outbreak personnel (administrative, operational) -+> Hunting activity -+> Humans acting as vectors -+> **ASF positive domestic pigs** --> Number of pig holdings in zoos -+> Collaboration of research institutions and zoos -+> Investments in vaccine research -+> Creativity -+> Availability of vaccine --> ASF positive cases in wild boar -+> Size of restriction zones I and II

164) **B** (13): Size of restriction zones I and II --> Number of domestic pig holdings --> Pressure on agriculture and food industry -+> Awareness of private investors -+> Money -+> Investments in vaccine research -+> Creativity -+> Availability of vaccine --> **ASF positive domestic pigs** -+> Outbreak personnel (administrative, operational) -+> Hunting activity -+> Sampling of wild boar -+> ASF positive cases in wild boar -+> Size of restriction zones I and II

165) **B** (13): Size of restriction zones I and II --> Number of domestic pig holdings --> Pressure on agriculture and food industry -+> Awareness of private investors -+> Money -+> Investments in vaccine research -+> Creativity -+> Availability of vaccine --> **ASF positive domestic pigs** -+> Outbreak personnel (administrative, operational) -+> Hunting activity -+> Humans acting as vectors -+> ASF positive cases in wild boar -+> Size of restriction zones I and II

166) **B** (13): Size of restriction zones I and II --> Number of domestic pig holdings --> Pressure on agriculture and food industry -+> Awareness of private investors -+> Money -+> Motivation -+> Outbreak personnel (administrative, operational) -+> Sampling of domestic pigs -+> **ASF positive domestic pigs** -+> Investments in vaccine research -+> Creativity -+> Availability of vaccine --> ASF positive cases in wild boar -+> Size of restriction zones I and II

167) **B** (13): Size of restriction zones I and II --> Number of domestic pig holdings --> Pressure on agriculture and food industry -+> Awareness of private investors -+> Money -+> Outbreak personnel (administrative, operational) -+> Hunting activity -+> Humans acting as vectors -+> **ASF positive domestic pigs** -+> Investments in vaccine research -+> Creativity -+> Availability of vaccine --> ASF positive cases in wild boar -+> Size of restriction zones I and II

168) **B** (13): Size of restriction zones I and II --> Number of pig holdings in zoos -+> Collaboration of research institutions and zoos -+> Investments in vaccine research -+> Creativity -+>

Availability of vaccine --> **ASF positive domestic pigs** --> Number of domestic pig holdings -+> Sampling of domestic pigs -+> Outbreak personnel (administrative, operational) -+> Hunting activity -+> Sampling of wild boar -+> **ASF positive cases in wild boar** -+> Size of restriction zones I and II

169) **B** (13): Size of restriction zones I and II --> Number of pig holdings in zoos -+> Collaboration of research institutions and zoos -+> Investments in vaccine research -+> Creativity -+> Availability of vaccine --> **ASF positive domestic pigs** --> Number of domestic pig holdings -+> Sampling of domestic pigs -+> Outbreak personnel (administrative, operational) -+> Hunting activity -+> Humans acting as vectors -+> **ASF positive cases in wild boar** -+> Size of restriction zones I and II

170) **B** (13): Size of restriction zones I and II --> Number of pig holdings in zoos --> Awareness of public/media -+> Awareness of politics -+> Money -+> Investments in vaccine research -+> Creativity -+> Availability of vaccine --> **ASF positive domestic pigs** -+> Outbreak personnel (administrative, operational) -+> Hunting activity -+> Sampling of wild boar -+> **ASF positive cases in wild boar** -+> Size of restriction zones I and II

171) **B** (13): Size of restriction zones I and II --> Number of pig holdings in zoos --> Awareness of public/media -+> Awareness of politics -+> Money -+> Investments in vaccine research -+> Creativity -+> Availability of vaccine --> **ASF positive domestic pigs** -+> Outbreak personnel (administrative, operational) -+> Hunting activity -+> Humans acting as vectors -+> **ASF positive cases in wild boar** -+> Size of restriction zones I and II

172) **B** (13): Size of restriction zones I and II --> Number of pig holdings in zoos --> Awareness of public/media -+> Awareness of politics -+> Money -+> Motivation -+> Outbreak personnel (administrative, operational) -+> Sampling of domestic pigs -+> **ASF positive domestic pigs** -+> Investments in vaccine research -+> Creativity -+> Availability of vaccine --> **ASF positive cases in wild boar** -+> Size of restriction zones I and II

173) **B** (13): Size of restriction zones I and II --> Number of pig holdings in zoos --> Awareness of public/media -+> Awareness of politics -+> Money -+> Outbreak personnel (administrative, operational) -+> Hunting activity -+> Humans acting as vectors -+> **ASF positive domestic pigs** -+> Investments in vaccine research -+> Creativity -+> Availability of vaccine --> **ASF positive cases in wild boar** -+> Size of restriction zones I and II

174) **B** (13): Size of restriction zones I and II --> Number of pig holdings in zoos --> Awareness of public/media -+> Awareness of private investors -+> Money -+> Investments in vaccine research -+> Creativity -+> Availability of vaccine --> **ASF positive domestic pigs** -+> Outbreak personnel (administrative, operational) -+> Hunting activity -+> Sampling of wild boar -+> **ASF positive cases in wild boar** -+> Size of restriction zones I and II

175) **B** (13): Size of restriction zones I and II --> Number of pig holdings in zoos --> Awareness of public/media -+> Awareness of private investors -+> Money -+> Investments in vaccine research -+> Creativity -+> Availability of vaccine --> **ASF positive domestic pigs** -+> Outbreak personnel (administrative, operational) -+> Hunting activity -+> Humans acting as vectors -+> **ASF positive cases in wild boar** -+> Size of restriction zones I and II

176) **B** (13): Size of restriction zones I and II --> Number of pig holdings in zoos --> Awareness of public/media -+> Awareness of private investors -+> Money -+> Motivation -+> Outbreak personnel (administrative, operational) -+> Sampling of domestic pigs -+> **ASF positive domestic pigs** -+> Investments in vaccine research -+> Creativity -+> Availability of vaccine --> **ASF positive cases in wild boar** -+> Size of restriction zones I and II

177) **B** (13): Size of restriction zones I and II --> Number of pig holdings in zoos --> Awareness of public/media -+> Awareness of private investors -+> Money -+> Outbreak personnel (administrative, operational) -+> Hunting activity -+> Humans acting as vectors -+> **ASF positive domestic pigs** -+> Investments in vaccine research -+> Creativity -+> Availability of vaccine --> **ASF positive cases in wild boar** -+> Size of restriction zones I and II

178) **R** (13): Collaboration of research institutions and zoos -+> Investments in vaccine research -+> Creativity -+> Availability of vaccine --> **ASF positive cases in wild boar** --> Wild boar population -+> Sampling of wild boar -+> Outbreak personnel (administrative, operational) -+> Education / Awareness raising -+> Qualification and awareness --> Humans acting as vectors -+> **ASF positive domestic pigs** --> Number of pig holdings in zoos -+> Collaboration of research institutions and zoos

179) **B** (13): Investments in vaccine research -+> Creativity -+> Availability of vaccine --> **ASF positive domestic pigs** --> Number of domestic pig holdings --> Pressure on agriculture and food industry -+> Awareness of private investors -+> Money -+> Motivation -+> Outbreak personnel (administrative, operational) -+> Hunting activity -+> Sampling of wild boar -+> **ASF positive cases in wild boar** -+> Investments in vaccine research

180) **B** (13): Investments in vaccine research -+> Creativity -+> Availability of vaccine --> **ASF positive domestic pigs** --> Number of domestic pig holdings --> Pressure on agriculture and food industry -+> Awareness of private investors -+> Money -+> Motivation -+> Outbreak personnel (administrative, operational) -+> Hunting activity -+> Humans acting as vectors -+> **ASF positive cases in wild boar** -+> Investments in vaccine research

181) **R** (13): Investments in vaccine research -+> Creativity -+> Availability of vaccine --> **ASF positive domestic pigs** --> Number of domestic pig holdings --> Pressure on agriculture and food industry -+> Awareness of private investors -+> Money -+> Outbreak personnel (administrative, operational) -+> Education / Awareness raising -+> Qualification and awareness --> Humans acting as vectors -+> **ASF positive cases in wild boar** -+> Investments in vaccine research

182) **R** (13): Investments in vaccine research -+> Creativity -+> Availability of vaccine --> **ASF positive domestic pigs** --> Number of domestic pig holdings --> Pressure on agriculture and food industry -+> Awareness of private investors -+> Money -+> Outbreak personnel (administrative, operational) -+> Hunting activity --> Wild boar population -+> Sampling of wild boar -+> **ASF positive cases in wild boar** -+> Investments in vaccine research

183) **B** (13): Investments in vaccine research -+> Creativity -+> Availability of vaccine --> **ASF positive domestic pigs** --> Number of pig holdings in zoos --> Awareness of public/media -+> Awareness of politics -+> Money -+> Motivation -+> Outbreak personnel (administrative, operational) -+> Hunting activity -+> Sampling of wild boar -+> **ASF positive cases in wild boar** -+> Investments in vaccine research

184) **B** (13): Investments in vaccine research -+> Creativity -+> Availability of vaccine --> **ASF positive domestic pigs** --> Number of pig holdings in zoos --> Awareness of public/media -+> Awareness of politics -+> Money -+> Motivation -+> Outbreak personnel (administrative, operational) -+> Hunting activity -+> Humans acting as vectors -+> **ASF positive cases in wild boar** -+> Investments in vaccine research

185) **R** (13): Investments in vaccine research -+> Creativity -+> Availability of vaccine --> **ASF positive domestic pigs** --> Number of pig holdings in zoos --> Awareness of public/media -+> Awareness of politics -+> Money -+> Outbreak personnel (administrative, operational) -+> Education /

Awareness raising -+> Qualification and awareness --> Humans acting as vectors -+> **ASF positive cases in wild boar** -+> Investments in vaccine research

186) **R** (13): Investments in vaccine research -+> Creativity -+> Availability of vaccine --> **ASF positive domestic pigs** --> Number of pig holdings in zoos --> Awareness of public/media -+> Awareness of politics -+> Money -+> Outbreak personnel (administrative, operational) -+> Hunting activity --> Wild boar population -+> Sampling of wild boar -+> **ASF positive cases in wild boar** -+> Investments in vaccine research

187) **B** (13): Investments in vaccine research -+> Creativity -+> Availability of vaccine --> **ASF positive domestic pigs** --> Number of pig holdings in zoos --> Awareness of public/media -+> Awareness of private investors -+> Money -+> Motivation -+> Outbreak personnel (administrative, operational) -+> Hunting activity -+> Sampling of wild boar -+> **ASF positive cases in wild boar** -+> Investments in vaccine research

188) **B** (13): Investments in vaccine research -+> Creativity -+> Availability of vaccine --> **ASF positive domestic pigs** --> Number of pig holdings in zoos --> Awareness of public/media -+> Awareness of private investors -+> Money -+> Motivation -+> Outbreak personnel (administrative, operational) -+> Hunting activity -+> Humans acting as vectors -+> **ASF positive cases in wild boar** -+> Investments in vaccine research

189) **R** (13): Investments in vaccine research -+> Creativity -+> Availability of vaccine --> **ASF positive domestic pigs** --> Number of pig holdings in zoos --> Awareness of public/media -+> Awareness of private investors -+> Money -+> Outbreak personnel (administrative, operational) -+> Education / Awareness raising -+> Qualification and awareness --> Humans acting as vectors -+> **ASF positive cases in wild boar** -+> Investments in vaccine research

190) **R** (13): Investments in vaccine research -+> Creativity -+> Availability of vaccine --> **ASF positive domestic pigs** --> Number of pig holdings in zoos --> Awareness of public/media -+> Awareness of private investors -+> Money -+> Outbreak personnel (administrative, operational) -+> Hunting activity --> Wild boar population -+> Sampling of wild boar -+> **ASF positive cases in wild boar** -+> Investments in vaccine research

191) **R** (13): Investments in vaccine research -+> Creativity -+> Availability of vaccine --> **ASF positive cases in wild boar** -+> Outbreak personnel (administrative, operational) -+> Education / Awareness raising -+> Qualification and awareness --> Humans acting as vectors -+> **ASF positive domestic pigs** --> Number of domestic pig holdings --> Pressure on agriculture and food industry -+> Awareness of private investors -+> Money -+> Investments in vaccine research

192) **R** (13): Investments in vaccine research -+> Creativity -+> Availability of vaccine --> **ASF positive cases in wild boar** -+> Outbreak personnel (administrative, operational) -+> Education / Awareness raising -+> Qualification and awareness --> Humans acting as vectors -+> **ASF positive domestic pigs** --> Number of pig holdings in zoos --> Awareness of public/media -+> Awareness of politics -+> Money -+> Investments in vaccine research

193) **R** (13): Investments in vaccine research -+> Creativity -+> Availability of vaccine --> **ASF positive cases in wild boar** -+> Outbreak personnel (administrative, operational) -+> Education / Awareness raising -+> Qualification and awareness --> Humans acting as vectors -+> **ASF positive domestic pigs** --> Number of pig holdings in zoos --> Awareness of public/media -+> Awareness of private investors -+> Money -+> Investments in vaccine research

194) **R** (13): Investments in vaccine research -+> Creativity -+> Availability of vaccine --> **ASF positive cases in wild boar** --> Wild boar population -+> Sampling of wild boar -+> Outbreak personnel (administrative, operational) -+> Sampling of domestic pigs -+> **ASF positive domestic pigs** -->

Number of domestic pig holdings --> Pressure on agriculture and food industry -+> Awareness of private investors -+> Money -+> Investments in vaccine research

195) **R** (13): Investments in vaccine research -+> Creativity -+> Availability of vaccine --> **ASF positive cases in wild boar** --> Wild boar population -+> Sampling of wild boar -+> Outbreak personnel (administrative, operational) -+> Sampling of domestic pigs -+> **ASF positive domestic pigs** --> Number of pig holdings in zoos --> Awareness of public/media -+> Awareness of politics -+> Money -+> Investments in vaccine research

196) **R** (13): Investments in vaccine research -+> Creativity -+> Availability of vaccine --> **ASF positive cases in wild boar** --> Wild boar population -+> Sampling of wild boar -+> Outbreak personnel (administrative, operational) -+> Sampling of domestic pigs -+> **ASF positive domestic pigs** --> Number of pig holdings in zoos --> Awareness of public/media -+> Awareness of private investors -+> Money -+> Investments in vaccine research

197) **R** (14): Size of restriction zones I and II --> Number of domestic pig holdings -+> Sampling of domestic pigs -+> **ASF positive domestic pigs** --> Number of pig holdings in zoos --> Awareness of public/media -+> Awareness of politics -+> Money -+> Motivation -+> Outbreak personnel (administrative, operational) -+> Education / Awareness raising -+> Qualification and awareness --> Humans acting as vectors -+> **ASF positive cases in wild boar** -+> Size of restriction zones I and II

198) **R** (14): Size of restriction zones I and II --> Number of domestic pig holdings -+> Sampling of domestic pigs -+> **ASF positive domestic pigs** --> Number of pig holdings in zoos --> Awareness of public/media -+> Awareness of politics -+> Money -+> Motivation -+> Outbreak personnel (administrative, operational) -+> Hunting activity --> Wild boar population -+> Sampling of wild boar -+> **ASF positive cases in wild boar** -+> Size of restriction zones I and II

199) **R** (14): Size of restriction zones I and II --> Number of domestic pig holdings -+> Sampling of domestic pigs -+> **ASF positive domestic pigs** --> Number of pig holdings in zoos --> Awareness of public/media -+> Awareness of private investors -+> Money -+> Motivation -+> Outbreak personnel (administrative, operational) -+> Education / Awareness raising -+> Qualification and awareness --> Humans acting as vectors -+> **ASF positive cases in wild boar** -+> Size of restriction zones I and II

200) **R** (14): Size of restriction zones I and II --> Number of domestic pig holdings -+> Sampling of domestic pigs -+> **ASF positive domestic pigs** --> Number of pig holdings in zoos --> Awareness of public/media -+> Awareness of private investors -+> Money -+> Motivation -+> Outbreak personnel (administrative, operational) -+> Hunting activity --> Wild boar population -+> Sampling of wild boar -+> **ASF positive cases in wild boar** -+> Size of restriction zones I and II

201) **R** (14): Size of restriction zones I and II --> Number of domestic pig holdings -+> Sampling of domestic pigs -+> Outbreak personnel (administrative, operational) -+> Education / Awareness raising -+> Qualification and awareness --> Humans acting as vectors -+> **ASF positive domestic pigs** --> Number of pig holdings in zoos -+> Collaboration of research institutions and zoos -+> Investments in vaccine research -+> Creativity -+> Availability of vaccine --> **ASF positive cases in wild boar** -+> Size of restriction zones I and II

202) **R** (14): Size of restriction zones I and II --> Number of domestic pig holdings --> Pressure on agriculture and food industry -+> Awareness of private investors -+> Money -+> Investments in vaccine research -+> Creativity -+> Availability of vaccine --> **ASF positive domestic pigs** -+> Outbreak personnel (administrative, operational) -+> Education / Awareness raising -+> Qualification and awareness --> Humans acting as vectors -+> **ASF positive cases in wild boar** -+> Size of restriction zones I and II

203) **R** (14): Size of restriction zones I and II --> Number of domestic pig holdings --> Pressure on agriculture and food industry -+> Awareness of private investors -+> Money -+> Investments in vaccine research -+> Creativity -+> Availability of vaccine --> **ASF positive domestic pigs** -+> Outbreak personnel (administrative, operational) -+> Hunting activity --> Wild boar population -+> Sampling of wild boar -+> **ASF positive cases in wild boar** -+> Size of restriction zones I and II

204) **B** (14): Size of restriction zones I and II --> Number of domestic pig holdings --> Pressure on agriculture and food industry -+> Awareness of private investors -+> Money -+> Motivation -+> Outbreak personnel (administrative, operational) -+> Hunting activity -+> Humans acting as vectors -+> **ASF positive domestic pigs** -+> Investments in vaccine research -+> Creativity -+> Availability of vaccine --> **ASF positive cases in wild boar** -+> Size of restriction zones I and II

205) **R** (14): Size of restriction zones I and II --> Number of domestic pig holdings --> Pressure on agriculture and food industry -+> Awareness of private investors -+> Money -+> Outbreak personnel (administrative, operational) -+> Sampling of domestic pigs -+> **ASF positive domestic pigs** --> Number of pig holdings in zoos -+> Collaboration of research institutions and zoos -+> Investments in vaccine research -+> Creativity -+> Availability of vaccine --> **ASF positive cases in wild boar** -+> Size of restriction zones I and II

206) **R** (14): Size of restriction zones I and II --> Number of domestic pig holdings --> Pressure on agriculture and food industry -+> Awareness of private investors -+> Money -+> Outbreak personnel (administrative, operational) -+> Education / Awareness raising -+> Qualification and awareness --> Humans acting as vectors -+> **ASF positive domestic pigs** -+> Investments in vaccine research -+> Creativity -+> Availability of vaccine --> **ASF positive cases in wild boar** -+> Size of restriction zones I and II

207) **R** (14): Size of restriction zones I and II --> Number of pig holdings in zoos -+> Collaboration of research institutions and zoos -+> Investments in vaccine research -+> Creativity -+> Availability of vaccine --> **ASF positive domestic pigs** --> Number of domestic pig holdings -+> Sampling of domestic pigs -+> Outbreak personnel (administrative, operational) -+> Education / Awareness raising -+> Qualification and awareness --> Humans acting as vectors -+> **ASF positive cases in wild boar** -+> Size of restriction zones I and II

208) **R** (14): Size of restriction zones I and II --> Number of pig holdings in zoos -+> Collaboration of research institutions and zoos -+> Investments in vaccine research -+> Creativity -+> Availability of vaccine --> **ASF positive domestic pigs** --> Number of domestic pig holdings -+> Sampling of domestic pigs -+> Outbreak personnel (administrative, operational) -+> Hunting activity --> Wild boar population -+> Sampling of wild boar -+> **ASF positive cases in wild boar** -+> Size of restriction zones I and II

209) **R** (14): Size of restriction zones I and II --> Number of pig holdings in zoos -+> Collaboration of research institutions and zoos -+> Investments in vaccine research -+> Creativity -+> Availability of vaccine --> **ASF positive domestic pigs** --> Number of domestic pig holdings --> Pressure on agriculture and food industry -+> Awareness of private investors -+> Money -+> Outbreak personnel (administrative, operational) -+> Sampling of wild boar -+> **ASF positive cases in wild boar** -+> Size of restriction zones I and II

210) **R** (14): Size of restriction zones I and II --> Number of pig holdings in zoos --> Awareness of public/media -+> Awareness of politics -+> Money -+> Investments in vaccine research -+> Creativity -+> Availability of vaccine --> **ASF positive domestic pigs** --> Number of domestic pig holdings -+> Sampling of domestic pigs -+> Outbreak personnel (administrative, operational) -

+> Sampling of wild boar -+> **ASF positive cases in wild boar** -+> Size of restriction zones I and II

211) **R** (14): Size of restriction zones I and II --> Number of pig holdings in zoos --> Awareness of public/media -+> Awareness of politics -+> Money -+> Investments in vaccine research -+> Creativity -+> Availability of vaccine --> **ASF positive domestic pigs** -+> Outbreak personnel (administrative, operational) -+> Education / Awareness raising -+> Qualification and awareness --> Humans acting as vectors -+> **ASF positive cases in wild boar** -+> Size of restriction zones I and II

212) **R** (14): Size of restriction zones I and II --> Number of pig holdings in zoos --> Awareness of public/media -+> Awareness of politics -+> Money -+> Investments in vaccine research -+> Creativity -+> Availability of vaccine --> **ASF positive domestic pigs** -+> Outbreak personnel (administrative, operational) -+> Hunting activity --> Wild boar population -+> Sampling of wild boar -+> **ASF positive cases in wild boar** -+> Size of restriction zones I and II

213) **B** (14): Size of restriction zones I and II --> Number of pig holdings in zoos --> Awareness of public/media -+> Awareness of politics -+> Money -+> Motivation -+> Outbreak personnel (administrative, operational) -+> Hunting activity -+> Humans acting as vectors -+> **ASF positive domestic pigs** -+> Investments in vaccine research -+> Creativity -+> Availability of vaccine --> **ASF positive cases in wild boar** -+> Size of restriction zones I and II

214) **R** (14): Size of restriction zones I and II --> Number of pig holdings in zoos --> Awareness of public/media -+> Awareness of politics -+> Money -+> Outbreak personnel (administrative, operational) -+> Education / Awareness raising -+> Qualification and awareness --> Humans acting as vectors -+> **ASF positive domestic pigs** -+> Investments in vaccine research -+> Creativity -+> Availability of vaccine --> **ASF positive cases in wild boar** -+> Size of restriction zones I and II

215) **R** (14): Size of restriction zones I and II --> Number of pig holdings in zoos --> Awareness of public/media -+> Awareness of private investors -+> Money -+> Investments in vaccine research -+> Creativity -+> Availability of vaccine --> **ASF positive domestic pigs** --> Number of domestic pig holdings -+> Sampling of domestic pigs -+> Outbreak personnel (administrative, operational) -+> Sampling of wild boar -+> **ASF positive cases in wild boar** -+> Size of restriction zones I and II

216) **R** (14): Size of restriction zones I and II --> Number of pig holdings in zoos --> Awareness of public/media -+> Awareness of private investors -+> Money -+> Investments in vaccine research -+> Creativity -+> Availability of vaccine --> **ASF positive domestic pigs** -+> Outbreak personnel (administrative, operational) -+> Education / Awareness raising -+> Qualification and awareness --> Humans acting as vectors -+> **ASF positive cases in wild boar** -+> Size of restriction zones I and II

217) **R** (14): Size of restriction zones I and II --> Number of pig holdings in zoos --> Awareness of public/media -+> Awareness of private investors -+> Money -+> Investments in vaccine research -+> Creativity -+> Availability of vaccine --> **ASF positive domestic pigs** -+> Outbreak personnel (administrative, operational) -+> Hunting activity --> Wild boar population -+> Sampling of wild boar -+> **ASF positive cases in wild boar** -+> Size of restriction zones I and II

218) **B** (14): Size of restriction zones I and II --> Number of pig holdings in zoos --> Awareness of public/media -+> Awareness of private investors -+> Money -+> Motivation -+> Outbreak personnel (administrative, operational) -+> Hunting activity -+> Humans acting as vectors -+> **ASF positive domestic pigs** -+> Investments in vaccine research -+> Creativity -+> Availability of vaccine --> **ASF positive cases in wild boar** -+> Size of restriction zones I and II

219) **R** (14): Size of restriction zones I and II --> Number of pig holdings in zoos --> Awareness of public/media -+> Awareness of private investors -+> Money -+> Outbreak personnel (administrative, operational) -+> Education / Awareness raising -+> Qualification and awareness --> Humans acting as vectors -+> **ASF positive domestic pigs** -+> Investments in vaccine research -+> Creativity -+> Availability of vaccine --> **ASF positive cases in wild boar** -+> Size of restriction zones I and II

220) **R** (14): Investments in vaccine research -+> Creativity -+> Availability of vaccine --> **ASF positive domestic pigs** --> Number of domestic pig holdings --> Pressure on agriculture and food industry -+> Awareness of private investors -+> Money -+> Motivation -+> Outbreak personnel (administrative, operational) -+> Education / Awareness raising -+> Qualification and awareness --> Humans acting as vectors -+> **ASF positive cases in wild boar** -+> Investments in vaccine research

221) **R** (14): Investments in vaccine research -+> Creativity -+> Availability of vaccine --> **ASF positive domestic pigs** --> Number of domestic pig holdings --> Pressure on agriculture and food industry -+> Awareness of private investors -+> Money -+> Motivation -+> Outbreak personnel (administrative, operational) -+> Hunting activity --> Wild boar population -+> Sampling of wild boar -+> **ASF positive cases in wild boar** -+> Investments in vaccine research

222) **R** (14): Investments in vaccine research -+> Creativity -+> Availability of vaccine --> **ASF positive domestic pigs** --> Number of pig holdings in zoos --> Awareness of public/media -+> Awareness of politics -+> Money -+> Motivation -+> Outbreak personnel (administrative, operational) -+> Education / Awareness raising -+> Qualification and awareness --> Humans acting as vectors -+> **ASF positive cases in wild boar** -+> Investments in vaccine research

223) **R** (14): Investments in vaccine research -+> Creativity -+> Availability of vaccine --> **ASF positive domestic pigs** --> Number of pig holdings in zoos --> Awareness of public/media -+> Awareness of politics -+> Money -+> Motivation -+> Outbreak personnel (administrative, operational) -+> Hunting activity --> Wild boar population -+> Sampling of wild boar -+> **ASF positive cases in wild boar** -+> Investments in vaccine research

224) **R** (14): Investments in vaccine research -+> Creativity -+> Availability of vaccine --> **ASF positive domestic pigs** --> Number of pig holdings in zoos --> Awareness of public/media -+> Awareness of private investors -+> Money -+> Motivation -+> Outbreak personnel (administrative, operational) -+> Education / Awareness raising -+> Qualification and awareness --> Humans acting as vectors -+> **ASF positive cases in wild boar** -+> Investments in vaccine research

225) **R** (14): Investments in vaccine research -+> Creativity -+> Availability of vaccine --> **ASF positive domestic pigs** --> Number of pig holdings in zoos --> Awareness of public/media -+> Awareness of private investors -+> Money -+> Motivation -+> Outbreak personnel (administrative, operational) -+> Hunting activity --> Wild boar population -+> Sampling of wild boar -+> **ASF positive cases in wild boar** -+> Investments in vaccine research

226) **R** (14): Investments in vaccine research -+> Creativity -+> Availability of vaccine --> **ASF positive cases in wild boar** --> Wild boar population -+> Sampling of wild boar -+> Outbreak personnel (administrative, operational) -+> Hunting activity -+> Humans acting as vectors -+> **ASF positive domestic pigs** --> Number of domestic pig holdings --> Pressure on agriculture and food industry -+> Awareness of private investors -+> Money -+> Investments in vaccine research

227) **R** (14): Investments in vaccine research -+> Creativity -+> Availability of vaccine --> **ASF positive cases in wild boar** --> Wild boar population -+> Sampling of wild boar -+> Outbreak personnel (administrative, operational) -+> Hunting activity -+> Humans acting as vectors -+> **ASF**

positive domestic pigs --> Number of pig holdings in zoos --> Awareness of public/media -+> Awareness of politics -+> Money -+> Investments in vaccine research

228) **R** (14): Investments in vaccine research -+> Creativity -+> Availability of vaccine --> **ASF positive cases in wild boar** --> Wild boar population -+> Sampling of wild boar -+> Outbreak personnel (administrative, operational) -+> Hunting activity -+> Humans acting as vectors -+> **ASF positive domestic pigs** --> Number of pig holdings in zoos --> Awareness of public/media -+> Awareness of private investors -+> Money -+> Investments in vaccine research

229) **R** (15): Size of restriction zones I and II --> Number of domestic pig holdings -+> Sampling of domestic pigs -+> Outbreak personnel (administrative, operational) -+> Hunting activity -+> Humans acting as vectors -+> **ASF positive domestic pigs** --> Number of pig holdings in zoos --> Awareness of public/media -+> Awareness of politics -+> Money -+> Investments in vaccine research -+> Creativity -+> Availability of vaccine --> **ASF positive cases in wild boar** -+> Size of restriction zones I and II

230) **R** (15): Size of restriction zones I and II --> Number of domestic pig holdings -+> Sampling of domestic pigs -+> Outbreak personnel (administrative, operational) -+> Hunting activity -+> Humans acting as vectors -+> **ASF positive domestic pigs** --> Number of pig holdings in zoos --> Awareness of public/media -+> Awareness of private investors -+> Money -+> Investments in vaccine research -+> Creativity -+> Availability of vaccine --> **ASF positive cases in wild boar** -+> Size of restriction zones I and II

231) **R** (15): Size of restriction zones I and II --> Number of domestic pig holdings --> Pressure on agriculture and food industry -+> Awareness of private investors -+> Money -+> Motivation -+> Outbreak personnel (administrative, operational) -+> Sampling of domestic pigs -+> **ASF positive domestic pigs** --> Number of pig holdings in zoos -+> Collaboration of research institutions and zoos -+> Investments in vaccine research -+> Creativity -+> Availability of vaccine --> **ASF positive cases in wild boar** -+> Size of restriction zones I and II

232) **R** (15): Size of restriction zones I and II --> Number of domestic pig holdings --> Pressure on agriculture and food industry -+> Awareness of private investors -+> Money -+> Motivation -+> Outbreak personnel (administrative, operational) -+> Education / Awareness raising -+> Qualification and awareness --> Humans acting as vectors -+> **ASF positive domestic pigs** -+> Investments in vaccine research -+> Creativity -+> Availability of vaccine --> **ASF positive cases in wild boar** -+> Size of restriction zones I and II

233) **R** (15): Size of restriction zones I and II --> Number of domestic pig holdings --> Pressure on agriculture and food industry -+> Awareness of private investors -+> Money -+> Outbreak personnel (administrative, operational) -+> Hunting activity -+> Humans acting as vectors -+> **ASF positive domestic pigs** --> Number of pig holdings in zoos -+> Collaboration of research institutions and zoos -+> Investments in vaccine research -+> Creativity -+> Availability of vaccine --> **ASF positive cases in wild boar** -+> Size of restriction zones I and II

234) **R** (15): Size of restriction zones I and II --> Number of pig holdings in zoos -+> Collaboration of research institutions and zoos -+> Investments in vaccine research -+> Creativity -+> Availability of vaccine --> **ASF positive domestic pigs** --> Number of domestic pig holdings --> Pressure on agriculture and food industry -+> Awareness of private investors -+> Money -+> Motivation -+> Outbreak personnel (administrative, operational) -+> Sampling of wild boar -+> **ASF positive cases in wild boar** -+> Size of restriction zones I and II

235) **R** (15): Size of restriction zones I and II --> Number of pig holdings in zoos -+> Collaboration of research institutions and zoos -+> Investments in vaccine research -+> Creativity -+> Availability of vaccine --> **ASF positive domestic pigs** --> Number of domestic pig holdings -->

Pressure on agriculture and food industry -+> Awareness of private investors -+> Money -+> Outbreak personnel (administrative, operational) -+> Hunting activity -+> Sampling of wild boar -+> ASF positive cases in wild boar -+> Size of restriction zones I and II

236) **R** (15): Size of restriction zones I and II --> Number of pig holdings in zoos -+> Collaboration of research institutions and zoos -+> Investments in vaccine research -+> Creativity -+> Availability of vaccine --> **ASF positive domestic pigs** --> Number of domestic pig holdings --> Pressure on agriculture and food industry -+> Awareness of private investors -+> Money -+> Outbreak personnel (administrative, operational) -+> Hunting activity -+> Humans acting as vectors -+> ASF positive cases in wild boar -+> Size of restriction zones I and II

237) **R** (15): Size of restriction zones I and II --> Number of pig holdings in zoos --> Awareness of public/media -+> Awareness of politics -+> Money -+> Investments in vaccine research -+> Creativity -+> Availability of vaccine --> **ASF positive domestic pigs** --> Number of domestic pig holdings -+> Sampling of domestic pigs -+> Outbreak personnel (administrative, operational) -+> Hunting activity -+> Sampling of wild boar -+> ASF positive cases in wild boar -+> Size of restriction zones I and II

238) **R** (15): Size of restriction zones I and II --> Number of pig holdings in zoos --> Awareness of public/media -+> Awareness of politics -+> Money -+> Investments in vaccine research -+> Creativity -+> Availability of vaccine --> **ASF positive domestic pigs** --> Number of domestic pig holdings -+> Sampling of domestic pigs -+> Outbreak personnel (administrative, operational) -+> Hunting activity -+> Humans acting as vectors -+> ASF positive cases in wild boar -+> Size of restriction zones I and II

239) **R** (15): Size of restriction zones I and II --> Number of pig holdings in zoos --> Awareness of public/media -+> Awareness of politics -+> Money -+> Motivation -+> Outbreak personnel (administrative, operational) -+> Education / Awareness raising -+> Qualification and awareness --> Humans acting as vectors -+> **ASF positive domestic pigs** -+> Investments in vaccine research -+> Creativity -+> Availability of vaccine --> ASF positive cases in wild boar -+> Size of restriction zones I and II

240) **R** (15): Size of restriction zones I and II --> Number of pig holdings in zoos --> Awareness of public/media -+> Awareness of private investors -+> Money -+> Investments in vaccine research -+> Creativity -+> Availability of vaccine --> **ASF positive domestic pigs** --> Number of domestic pig holdings -+> Sampling of domestic pigs -+> Outbreak personnel (administrative, operational) -+> Hunting activity -+> Sampling of wild boar -+> ASF positive cases in wild boar -+> Size of restriction zones I and II

241) **R** (15): Size of restriction zones I and II --> Number of pig holdings in zoos --> Awareness of public/media -+> Awareness of private investors -+> Money -+> Investments in vaccine research -+> Creativity -+> Availability of vaccine --> **ASF positive domestic pigs** --> Number of domestic pig holdings -+> Sampling of domestic pigs -+> Outbreak personnel (administrative, operational) -+> Hunting activity -+> Humans acting as vectors -+> ASF positive cases in wild boar -+> Size of restriction zones I and II

242) **R** (15): Size of restriction zones I and II --> Number of pig holdings in zoos --> Awareness of public/media -+> Awareness of private investors -+> Money -+> Motivation -+> Outbreak personnel (administrative, operational) -+> Education / Awareness raising -+> Qualification and awareness --> Humans acting as vectors -+> **ASF positive domestic pigs** -+> Investments in vaccine research -+> Creativity -+> Availability of vaccine --> ASF positive cases in wild boar -+> Size of restriction zones I and II

243) **B** (15): Investments in vaccine research -+> Creativity -+> Availability of vaccine --> **ASF positive cases in wild boar** --> Wild boar population -+> Sampling of wild boar -+> Outbreak personnel (administrative, operational) -+> Education / Awareness raising -+> Qualification and awareness --> Humans acting as vectors -+> **ASF positive domestic pigs** --> Number of domestic pig holdings --> Pressure on agriculture and food industry -+> Awareness of private investors -+> Money -+> Investments in vaccine research

244) **B** (15): Investments in vaccine research -+> Creativity -+> Availability of vaccine --> **ASF positive cases in wild boar** --> Wild boar population -+> Sampling of wild boar -+> Outbreak personnel (administrative, operational) -+> Education / Awareness raising -+> Qualification and awareness --> Humans acting as vectors -+> **ASF positive domestic pigs** --> Number of pig holdings in zoos --> Awareness of public/media -+> Awareness of politics -+> Money -+> Investments in vaccine research

245) **B** (15): Investments in vaccine research -+> Creativity -+> Availability of vaccine --> **ASF positive cases in wild boar** --> Wild boar population -+> Sampling of wild boar -+> Outbreak personnel (administrative, operational) -+> Education / Awareness raising -+> Qualification and awareness --> Humans acting as vectors -+> **ASF positive domestic pigs** --> Number of pig holdings in zoos --> Awareness of public/media -+> Awareness of private investors -+> Money -+> Investments in vaccine research

246) **B** (16): Size of restriction zones I and II --> Number of domestic pig holdings -+> Sampling of domestic pigs -+> Outbreak personnel (administrative, operational) -+> Education / Awareness raising -+> Qualification and awareness --> Humans acting as vectors -+> **ASF positive domestic pigs** --> Number of pig holdings in zoos --> Awareness of public/media -+> Awareness of politics -+> Money -+> Investments in vaccine research -+> Creativity -+> Availability of vaccine --> **ASF positive cases in wild boar** -+> Size of restriction zones I and II

247) **B** (16): Size of restriction zones I and II --> Number of domestic pig holdings -+> Sampling of domestic pigs -+> Outbreak personnel (administrative, operational) -+> Education / Awareness raising -+> Qualification and awareness --> Humans acting as vectors -+> **ASF positive domestic pigs** --> Number of pig holdings in zoos --> Awareness of public/media -+> Awareness of private investors -+> Money -+> Investments in vaccine research -+> Creativity -+> Availability of vaccine --> **ASF positive cases in wild boar** -+> Size of restriction zones I and II

248) **R** (16): Size of restriction zones I and II --> Number of domestic pig holdings --> Pressure on agriculture and food industry -+> Awareness of private investors -+> Money -+> Motivation -+> Outbreak personnel (administrative, operational) -+> Hunting activity -+> Humans acting as vectors -+> **ASF positive domestic pigs** --> Number of pig holdings in zoos -+> Collaboration of research institutions and zoos -+> Investments in vaccine research -+> Creativity -+> Availability of vaccine --> **ASF positive cases in wild boar** -+> Size of restriction zones I and II

249) **B** (16): Size of restriction zones I and II --> Number of domestic pig holdings --> Pressure on agriculture and food industry -+> Awareness of private investors -+> Money -+> Outbreak personnel (administrative, operational) -+> Education / Awareness raising -+> Qualification and awareness --> Humans acting as vectors -+> **ASF positive domestic pigs** --> Number of pig holdings in zoos -+> Collaboration of research institutions and zoos -+> Investments in vaccine research -+> Creativity -+> Availability of vaccine --> **ASF positive cases in wild boar** -+> Size of restriction zones I and II

250) **R** (16): Size of restriction zones I and II --> Number of pig holdings in zoos -+> Collaboration of research institutions and zoos -+> Investments in vaccine research -+> Creativity -+> Availability of vaccine --> **ASF positive domestic pigs** --> Number of domestic pig holdings -->

Pressure on agriculture and food industry -+> Awareness of private investors -+> Money -+> Motivation -+> Outbreak personnel (administrative, operational) -+> Hunting activity -+> Sampling of wild boar -+> **ASF positive cases in wild boar** -+> Size of restriction zones I and II

251) **R** (16): Size of restriction zones I and II --> Number of pig holdings in zoos -+> Collaboration of research institutions and zoos -+> Investments in vaccine research -+> Creativity -+> Availability of vaccine --> **ASF positive domestic pigs** --> Number of domestic pig holdings --> Pressure on agriculture and food industry -+> Awareness of private investors -+> Money -+> Motivation -+> Outbreak personnel (administrative, operational) -+> Hunting activity -+> Humans acting as vectors -+> **ASF positive cases in wild boar** -+> Size of restriction zones I and II

252) **B** (16): Size of restriction zones I and II --> Number of pig holdings in zoos -+> Collaboration of research institutions and zoos -+> Investments in vaccine research -+> Creativity -+> Availability of vaccine --> **ASF positive domestic pigs** --> Number of domestic pig holdings --> Pressure on agriculture and food industry -+> Awareness of private investors -+> Money -+> Outbreak personnel (administrative, operational) -+> Education / Awareness raising -+> Qualification and awareness --> Humans acting as vectors -+> **ASF positive cases in wild boar** -+> Size of restriction zones I and II

253) **B** (16): Size of restriction zones I and II --> Number of pig holdings in zoos -+> Collaboration of research institutions and zoos -+> Investments in vaccine research -+> Creativity -+> Availability of vaccine --> **ASF positive domestic pigs** --> Number of domestic pig holdings --> Pressure on agriculture and food industry -+> Awareness of private investors -+> Money -+> Outbreak personnel (administrative, operational) -+> Hunting activity --> Wild boar population -+> Sampling of wild boar -+> **ASF positive cases in wild boar** -+> Size of restriction zones I and II

254) **B** (16): Size of restriction zones I and II --> Number of pig holdings in zoos --> Awareness of public/media -+> Awareness of politics -+> Money -+> Investments in vaccine research -+> Creativity -+> Availability of vaccine --> **ASF positive domestic pigs** --> Number of domestic pig holdings -+> Sampling of domestic pigs -+> Outbreak personnel (administrative, operational) -+> Education / Awareness raising -+> Qualification and awareness --> Humans acting as vectors -+> **ASF positive cases in wild boar** -+> Size of restriction zones I and II

255) **B** (16): Size of restriction zones I and II --> Number of pig holdings in zoos --> Awareness of public/media -+> Awareness of politics -+> Money -+> Investments in vaccine research -+> Creativity -+> Availability of vaccine --> **ASF positive domestic pigs** --> Number of domestic pig holdings -+> Sampling of domestic pigs -+> Outbreak personnel (administrative, operational) -+> Hunting activity --> Wild boar population -+> Sampling of wild boar -+> **ASF positive cases in wild boar** -+> Size of restriction zones I and II

256) **B** (16): Size of restriction zones I and II --> Number of pig holdings in zoos --> Awareness of public/media -+> Awareness of private investors -+> Money -+> Investments in vaccine research -+> Creativity -+> Availability of vaccine --> **ASF positive domestic pigs** --> Number of domestic pig holdings -+> Sampling of domestic pigs -+> Outbreak personnel (administrative, operational) -+> Education / Awareness raising -+> Qualification and awareness --> Humans acting as vectors -+> **ASF positive cases in wild boar** -+> Size of restriction zones I and II

257) **B** (16): Size of restriction zones I and II --> Number of pig holdings in zoos --> Awareness of public/media -+> Awareness of private investors -+> Money -+> Investments in vaccine research -+> Creativity -+> Availability of vaccine --> **ASF positive domestic pigs** --> Number of domestic pig holdings -+> Sampling of domestic pigs -+> Outbreak personnel (administrative,

operational) -+> Hunting activity --> Wild boar population -+> Sampling of wild boar -+> ASF positive cases in wild boar -+> Size of restriction zones I and II

258) **B** (17): Size of restriction zones I and II --> Number of domestic pig holdings --> Pressure on agriculture and food industry -+> Awareness of private investors -+> Money -+> Motivation -+> Outbreak personnel (administrative, operational) -+> Education / Awareness raising -+> Qualification and awareness --> Humans acting as vectors -+> **ASF positive domestic pigs** --> Number of pig holdings in zoos -+> Collaboration of research institutions and zoos -+> Investments in vaccine research -+> Creativity -+> Availability of vaccine --> ASF positive cases in wild boar -+> Size of restriction zones I and II

259) **B** (17): Size of restriction zones I and II --> Number of pig holdings in zoos -+> Collaboration of research institutions and zoos -+> Investments in vaccine research -+> Creativity -+> Availability of vaccine --> **ASF positive domestic pigs** --> Number of domestic pig holdings --> Pressure on agriculture and food industry -+> Awareness of private investors -+> Money -+> Motivation -+> Outbreak personnel (administrative, operational) -+> Education / Awareness raising -+> Qualification and awareness --> Humans acting as vectors -+> ASF positive cases in wild boar -+> Size of restriction zones I and II

260) **B** (17): Size of restriction zones I and II --> Number of pig holdings in zoos -+> Collaboration of research institutions and zoos -+> Investments in vaccine research -+> Creativity -+> Availability of vaccine --> **ASF positive domestic pigs** --> Number of domestic pig holdings --> Pressure on agriculture and food industry -+> Awareness of private investors -+> Money -+> Motivation -+> Outbreak personnel (administrative, operational) -+> Hunting activity --> Wild boar population -+> Sampling of wild boar -+> ASF positive cases in wild boar -+> Size of restriction zones I and II
